# Supplementary material for: A microbial supply chain for production of the anti-cancer drug vinblastine
Source: Nature. 2022 Aug 31;609(7926):341–7. doi: 10.1038/s41586-022-05157-3 (PMC9452304; doi:10.1038/s41586-022-05157-3)
Supplement: Supplementary file 1 — Supplementary Tables 1–8. Lists of heterologous genes for vinblastine biosynthesis; yeast strains; sequences; plasmids; chemicals; analytical standards; MS retention times and standard mixture concentrations. Supplementary Methods. Method descriptions for purification of catharanthine and vindoline, as well as descriptions of analytical methods. Supplementary Figs. 1–12. Data describing the subcellular localization of enzymes; metabolite production; functional expression of pathway modules; specific growth rates of yeast strains and chromatograms. [file 41586_2022_5157_MOESM1_ESM.pdf]

nature portfolio

https://doi.org/10.1038/s41586-022-05157-3

Supplementary information

A microbial supply chain for production of the anti-cancer drug vinblastine

In the format provided by the
authors and unedited

Supplementary Material

A microbial supply chain for production of anti-cancer drug vinblastine

Jie Zhang[1], Lea G. Hansen[1], Olga Gudich[1], Konrad Viehrig[1], Lærke M. M. Lassen[1], Lars Schrübbers[1], Khem Bahadur Adhikari[1], Paulina Rubaszka[1], Elena Carrasquer-Alvarez[1], Ling Chen[1], Vasil D'Ambrosio[1], Beata Lehka[1], Ahmad Kasem Haidar[1], Saranya Nallapareddy[1], Konstantina Giannakou[1], Marcos Laloux[1], Dushica Arsovska[1], Marcus A. K. Jørgensen[1], Leanne Jade G. Chan[2,3], Mette Kristensen[1], Hanne B. Christensen[1], Suresh Sudarsan[1], Emily A. Stander[4], Edward Baidoo[2,3], Christopher J. Petzold[2,3], Tune Wulff[1], Sarah O'Connor[5], Vincent Courdavault[4], Michael K. Jensen[1,*], and Jay D. Keasling[1-3,6,7,*]

[1] Novo Nordisk Foundation Center for Biosustainability, Technical University of Denmark, Kgs. Lyngby, Denmark

[2] Joint BioEnergy Institute, Emeryville, CA, USA

[3] Biological Systems and Engineering Division, Lawrence Berkeley National Laboratory, Berkeley, CA, USA

[4] EA2106 Biomolécules et Biotechnologies Végétales, Université de Tours, F-37200, Tours, France

[5] Department of Natural Product Biosynthesis, Max Planck Institute for Chemical Ecology, D-07745 Jena, Germany

[6] Department of Chemical and Biomolecular Engineering, Department of Bioengineering, University of California, Berkeley, CA, USA

[7] Center for Synthetic Biochemistry, Institute for Synthetic Biology, Shenzhen Institutes of Advanced Technologies, Shenzhen, China

Supplementary Tables

Supplementary Table 1. List of heterologous genes for vinblastine biosynthesis

Enzyme	Origin	Short Name	GenBank or reference
geranyl diphosphate synthase	*Abies grandis*	*Agr*GPPS2	AAN01134.1
farnesyl pyrophosphate synthase variant N144W	*Gallus gallus*	*Gga*FPS^{N144W}	P08836.2
geraniol synthase	*Catharanthus roseus*	*Cro*GES	AFD64744.1
NADPH-cytochrome P450 reductase	*Catharanthus roseus*	*Cro*CPR	CAA49446.1
cytochrome b5	*Catharanthus roseus*	*Cro*CYB5	A0A0C5DKP2
geraniol 8-hydroxylase	*Catharanthus roseus*	*Cro*G8H	CAC80883.1
8-hydroxygeraniol oxidoreductase, type A (NAD^+-dependent)	*Catharanthus roseus*	*Cro*8HGO-A	AHK60836.1
	Vinca minor	*Vmi*8HGO-A	Stander et al., 2020
8-hydroxygeraniol dehydrogenase, ($NADP^+$-dependent)	*Catharanthus roseus*	*Cro*8HGO-B	AAQ55962.1
	Rauvolfia tetraphylla	*Rte*8HGO-B	gnl|onekp|QEHE_scaffold_2015271
	Sesamum indicum	*Sin*8HGO-B	XP_011097456.1
	Populus trichocarpa	*Ptr*8HGO-B	XP_002322822.1
iridoid synthase	*Catharanthus roseus*	*Cro*ISY	AFW98981.1
	Nepeta mussinii	*Nmu*ISY	KY882236.1
	Nepeta cataria	*Nca*ISY	KY882234.1
	Olea europae	*Oeu*ISY	KT954038.1
	Digitalis purpurea	*Dpu*ISY	ACZ66261.1
iridoid cyclase	*Nepeta mussinii*	*Nmu*NEPS2	AXF35972.1
	Nepeta cataria	*Nca*MLPLA	QKE59462.1
	Nepeta cataria	*Nca*MLPLB	QKE59461.1
	Nepeta mussinii	*Nmu*MLPL	QKE59448.1
iridoid oxidase	*Catharanthus roseus*	*Cro*IO	AHK60833.1
alcohol dehydrogenase 2	*Catharanthus roseus*	*Cro*CYPADH	KP411012.1
7-deoxyloganetic acid glucosyl transferase	*Catharanthus roseus*	*Cro*7DLGT	AHK60835.1
7-deoxyloganic acid hydroxylase	*Catharanthus roseus*	*Cro*7DLH	AHK60834.1
loganic acid O-methyltransferase	*Catharanthus roseus*	*Cro*LAMT	ABW38009.1
secologanin synthase	*Catharanthus roseus*	*Cro*SLS	AKF02529.1
tryptophan decarboxylase	*Catharanthus roseus*	*Cro*TDC	CAA47898.1

strictosidine synthase	*Catharanthus roseus*	*Cro*STR	CAA43936.1
	Rauvolfia serpentina	*Rse*STR	CAA68725.1
	Ophiorrhiza pumila	*Opu*STR	BAB47180.1
	Morus notabilis	*Mno*STR	EXC25148.1
	Arabidopsis thaliana	*Ath*STR	AAB40594.1
	Camptotheca acuminata	*Cac*STR	AES93117.1
	Tabernaemontana elegans	*Tel*STR	AEY82398.1
strictosidine-O-β-D-glucosidase homolog	*Actinidia chinensis var. chinensis*	*Ach*GD1	PSR88404.1
	Actinidia chinensis var. chinensis	*Ach*GD2	PSR90224.1
	Actinidia chinensis var. chinensis	*Ach*GD3	PSS10019.1
	Amsonia hubrichtii	*Ahu*SGD-like	MAGPIE:poc_POCLF1PC_ Velvet--Contig238
	Camptotheca acuminata	*Cac*SGD	AES93119.1
	Carapichea ipecacuanha	*Ipe*Glu1	BAH02544.1_1
	Catharanthus roseus	*Cro*SGD	ABW77570.1
	Chelidonium majus	*Cma*GD	MAGPIE:chm_CMART2PF _Velvet--Singlet14063
	Coffea arabica	*Car*RGD-like	XP_027073002.1
	Coffea eugenioides	*Ceu*RGD-like	XP_027174620.1
	Erythranthe guttata	*Egu*GD	XP_012830199.1
	Exacum affine	*Eaf*GD	onekp:KPUM_scaffold_2 102222
	Gelsemium sempervirens	*Gse*SGD	AXK92564.1
	Glycine soja	*Gso*GD	RZB79711.1
	Handroanthus impetiginosus	*Him*GD1	PIN02484.1
	Handroanthus impetiginosus	*Him*GD2	PIN06789.1
	Handroanthus impetiginosus	*Him*GD3	PIN07435.1
	Handroanthus impetiginosus	*Him*GD4	PIN20653.1
	Helianthus annuus	*Han*GD	XP_022001174.1

	Helianthus annuus	*Han*SGD-like	XP_02201531.1
	Heliocybe sulcata	*Hsu*GD	TFK52902.1
	Hydnomerulius pinastri MD-312	*Hpi*GD	KIJ63193.1
	Ipomoea nil	*Ini*RGD-like	XP_019170759.1
	Lactuca sativa	*Lsa*GD	XP_023770227.1
	Lactuca sativa	*Lsa*RGD-like	XP_023728599.1
	Lomentospora prolificans	*Lpr*GD	PKS11920.1
	Lonicera japonica Thunb.	*Lja*GD1	MAGPIE:lja_LJAFL1VD_Velvet--Contig12159
	Lonicera japonica Thunb.	*Lja*GD2	MAGPIE:lja_LJAFL1VD_assembled--LJAFL1VD_rep_c920
	Madurella mycetomatis	*Mmy*GD	KXX74348.1
	Moniliophthora roreri MCA 2997	*Mro*GD	ESK96275.1
	Nicotiana tabacum	*Nta*VGD-like	XP_016502839.1
	Nyssa sinensis	*Nsi*GD1	KAA8549635.1
	Nyssa sinensis	*Nsi*GD2	KAA8549636.1
	Olea europaea	*Oeu*GD	XP_022888763.1
	Olea europaea	*Oeu*RGD-like1	XP_022888219.1_1
	Ophiorrhiza pumila	*Opu*GD	BAP90523.1_1
	Pyricularia grisea	*Pgr*GD	AAX07701.1
	Rauvolfia serpentina	*Rse*SGD	CAC83098.1
	Rauvolfia serpentina	*Rse*RGD	AAF03675.1_1
	Rauvolfia verticillata	*Rve*SGD	AFI71457.1
	Salvia splendens	*Ssp*GD1	TEY12393.1
	Salvia splendens	*Ssp*GD2	TEY66597.1
	Scedosporium apiospermum	*Sap*SGD	KEZ44838.1
	Sesamum indicum	*Sin*GD	XP_011094151.1
	Tabernaemontana elegans	*Tel*SGD-like	MAGPIE:tel_TELWL1VD_velvet1--Contig26920
	Uncaria tomentosa	*Uto*SGD	AFN69080.1
	Vigna unguiculata	*Vun*CGD-like	XP_027910736.1
	Vinca minor	*Vmi*RG-like	MAGPIE:vmi_VMIWL1VD_Trinity--comp21849_c0_seq1

	Vinca minor	*Vmi*SGD-like1	MAGPIE:vmi_VMIWL1VD_Trinity--comp323_c0_seq1
	Vinca minor	*Vmi*SGD-like2	MAGPIE:vmi_VMIWL1VD_assembled--VMIWL1VD_rep_c1191
	Vinca minor	*Vmi*SGD-like3	MAGPIE:vmi_VMIWL1VD_assembled--VMIWL1VD_rep_c247
tetrahydroalstonine synthase	*Catharanthus roseus*	*Cro*THAS	KM524258
heteroyohimbine synthase	*Catharanthus roseus*	*Cro*HYS	KU865325.1
geissoschizine synthase	*Catharanthus roseus*	*Cro*GS	AHK60846.1
geissoschizine oxidase	*Catharanthus roseus*	*Cro*GO	AHK60839.1
protein Redox 1	*Catharanthus roseus*	*Cro*Redox1	AVM85917.1
protein Redox 2	*Catharanthus roseus*	*Cro*Redox2	AVM85918.1
stemmadenine-O-acetyltransferase	*Catharanthus roseus*	*Cro*SAT	AVM85919.1
O-acetylstemmadenine oxidase precondylocarpine acetate synthase	*Catharanthus roseus*	*Cro*PAS	AYE56095.1
dihydroprecondylocarpine acetate synthase	*Catharanthus roseus*	*Cro*DPAS	ANQ45231.1
catharanthine synthase	*Catharanthus roseus*	*Cro*CS	AVM85920.1
tabersonine synthase	*Catharanthus roseus*	*Cro*TS	AVM85921.1
tabersonine 16-hydroxylase 1	*Catharanthus roseus*	*Cro*T16H1	ACM92061.1
tabersonine 16-hydroxylase 2	*Catharanthus roseus*	*Cro*T16H2	AEB69788.1
tabersonine 16-O-methyltransferase	*Catharanthus roseus*	*Cro*16OMT	ABR20103.1
tabersonine 3-oxygenase	*Catharanthus roseus*	*Cro*T3O	AEX07771.1
16-methoxy-2,3-dihydro-3-hydroxytabersonine synthase	*Catharanthus roseus*	*Cro*T3R	AKV60044.1
3-hydroxy-16-methoxy-2,3-dihydrotabersonine N-methyltransferase	*Catharanthus roseus*	*Cro*NMT	AHH33092.1
deacetoxyvindoline 4-hydroxylase	*Catharanthus roseus*	*Cro*D4H	AAB97311.1
deacetylvindoline O-acetyltransferase	*Catharanthus roseus*	*Cro*DAT	AAC99311.1
peroxidase	*Catharanthus roseus*	*Cro*PRX1	CAJ84723.1

Notes:

*Agr*GPPS2 from the grand fir (*Abies grandis*) is a specific geranyl diphosphate synthase (GPPS)[1] that does not produce farnesyl pyrophosphate. The *Gga*FPS N144W variant (*Gga*FPS^{N144W}) had discriminated binding towards GPP as the substrate for the second reaction catalyzed by the enzyme[2].

The yeast native farnesyl pyrophosphate synthetase (encoded by *ERG20*) catalyses the formation of farnesyl pyrophosphate (FPP), while the double mutant F96W-N127W variant was reported to have abolished FPPS activity and substantially improved monoterpene production in engineered yeast[3,4].

We combined all these strategies, together with the knock-down of native *ERG20*, to ensure a strong conversion of DMAPP and IPP into GPP, while maintaining a relatively low production of FPP that is essential as it's required for ergosterol biosynthesis.

Supplementary Table 2. List of yeast strains

Name		Note	Reference
CEN.PK2-1C	MAT**a**, his3D1, leu2-3_112, ura3-52, trp1-289	Wild-type strain	EUROSCARF
MIA-AU	MAT**a**, his3D1, leu2-3_112, ura3-52, trp1-289, atf1Δ oye2Δ, P_{TEF1}-CroCPR-T_{PRM9}, P_{PGK1}-CroCYB5-T_{VPS13}, P_{PGK1}-CroTDC-T_{PRM9}, P_{TEF1}-AgrGPPS2-T_{VPS13}, P_{FBA1}-GgaFPS^{N144W}-T_{IDP1}, P_{REV1}-ERG20-T_{ERG20}, P_{TDH3}-tHMG1-T_{ADH1}, P_{PGK1}-tCroGES-T_{VPS13}, P_{TDH3}-CroG8H-T_{ADH1}, P_{FBA1}-Cro8HGO-B-T_{IDP1}, P_{FBA1}-CroISY-T_{IDP1}, P_{TEF1}-CroIO-T_{PRM9}, P_{TEF1}-CroCYPADH-T_{CYC1}, P_{PGK1}-Cro7DLGT-T_{VPS13}, P_{TDH3}-Cro7DLH-T_{ADH1}, P_{TDH3}-CroLAMT-T_{ADH1}, P_{FBA1}-CroSLS-T_{IDP1}, P_{TEF1}-tCroSTR-T_{PRM9}	*de novo* strictosidine	This study
MIA-AW-2	MAT**a**, his3D1, leu2-3_112, ura3-52, trp1-289, atf1Δ oye2Δ oye3Δ ari1Δ, P_{TEF1}-CroCPR-T_{PRM9}, P_{PGK1}-CroCYB5-T_{VPS13}, P_{PGK1}-CroTDC-T_{PRM9}, P_{TEF1}-AgrGPPS2-T_{VPS13}, P_{FBA1}-GgaFPS^{N144W}-T_{IDP1}, P_{REV1}-ERG20-T_{ERG20}, P_{TDH3}-tHMG1-T_{ADH1}, P_{PGK1}-tCroGES-T_{VPS13}, P_{TDH3}-CroG8H-T_{ADH1}, P_{FBA1}-Cro8HGO-B-T_{IDP1}, P_{FBA1}-CroISY-T_{IDP1}, P_{TEF1}-CroIO-T_{PRM9}, P_{TEF1}-CroCYPADH-T_{CYC1}, P_{PGK1}-Cro7DLGT-T_{VPS13}, P_{TDH3}-Cro7DLH-T_{ADH1}, P_{TDH3}-CroLAMT-T_{ADH1}, P_{FBA1}-CroSLS-T_{IDP1}, P_{TEF1}-tCroSTR-T_{PRM9},	*de novo* strictosidine	This study
MIA-BC	MAT**a**, his3D1, leu2-3_112, ura3-52, trp1-289, atf1Δ oye2Δ, P_{TEF1}-CroCPR-T_{PRM9}, P_{PGK1}-CroCYB5-T_{VPS13}, P_{PGK1}-CroTDC-T_{PRM9}, P_{TEF1}-AgrGPPS2-T_{VPS13}, P_{FBA1}-GgaFPS^{N144W}-T_{IDP1}, pREV1-ERG20-(KD), P_{TDH3}-tHMG1-T_{ADH1}, P_{PGK1}-tCroGES-T_{VPS13}, P_{TDH3}-CroG8H-T_{ADH1}, P_{FBA1}-Cro8HGO-B-T_{IDP1}, P_{FBA1}-CroISY-T_{IDP1}, P_{TEF1}-CroIO-T_{PRM9}, P_{TEF1}-CroCYPADH-T_{CYC1}, P_{PGK1}-Cro7DLGT-T_{VPS13}, P_{TDH3}-Cro7DLH-T_{ADH1}, P_{TDH3}-CroLAMT-T_{ADH1}, P_{FBA1}-CroSLS-T_{IDP1}	*de novo* secologanin	This study
MIA-B-0	MAT**a**, his3D1, leu2-3_112, ura3-52, trp1-289, P_{TEF1}-SpyCas9-T_{CYC1}	*Spy*Cas9 integrated into the wild-type strain	This study
MIA-BB	MAT**a**, his3D1, leu2-3_112, ura3-52, trp1-289, atf1Δ oye2Δ, P_{TEF1}-SpyCas9-T_{CYC1},	Knockout shunt pathway	This study
MIA-BE	MAT**a**, his3D1, leu2-3_112, ura3-52, trp1-289, atf1Δ oye2Δ adh6Δ oye3Δ ari1Δ, P_{TEF1}-SpyCas9-T_{CYC1}	Knockout shunt pathway	This study
MIA-BG	MAT**a**, his3D1, leu2-3_112, ura3-52, trp1-289, atf1Δ oye2Δ adh6Δ oye3Δ ari1Δ, P_{TEF1}-SpyCas9-T_{CYC1}, P_{TEF1}-CroCPR-T_{PRM9}, P_{PGK1}-CroCYB5-T_{IDP1}, P_{TDH3}-CroG8H-T_{ADH1}, P_{TPI1}-Cro8HGO-B-T_{IDP1}, P_{FBA1}-CroISY-T_{CPS1}, P_{TEF2}-CroIO-T_{CYC1}, P_{FBA1}-CroCYPADH-T_{CPS1}, P_{PGK1}-Cro7DLGT-T_{VPS13}, P_{TEF2}-Cro7DLH-T_{CYC1}, P_{TDH3}-CroLAMT-T_{ADH1}, P_{TPI1}-CroSLS-T_{IDP1}, P_{TEF1}-tCroSTR-T_{PRM9}	strictosidine (from geraniol and tryptamine feeding)	This study

MIA-BJ	MAT**a**, his3D1, leu2-3_112, ura3-52, trp1-289, atf1Δ oye2Δ adh6Δ oye3Δ ari1Δ, P$_{TEF1}$-SpyCas9-T$_{CYC1}$, P$_{TEF1}$-CroCPR-T$_{PRM9}$, P$_{PGK1}$-CroCYB5-T$_{IDP1}$, P$_{TDH3}$-CroG8H-T$_{ADH1}$, P$_{TPI1}$-Cro8HGO-B-T$_{IDP1}$, P$_{FBA1}$-CroISY-T$_{CPS1}$, P$_{TEF2}$-CroIO-T$_{CYC1}$, P$_{FBA1}$-CroCYPADH-T$_{CPS1}$, P$_{PGK1}$-Cro7DLGT-T$_{VPS13}$, P$_{TEF2}$-Cro7DLH-T$_{CYC1}$, P$_{TDH3}$-CroLAMT-T$_{ADH1}$, P$_{TPI1}$-CroSLS-T$_{IDP1}$, P$_{FBA1}$-CroSTR-T$_{PRM9}$	strictosidine (from geraniol and tryptamine feeding)	This study
MIA-BKV-1	MAT**a**, his3D1, leu2-3_112, ura3-52, trp1-289, atf1Δ oye2Δ adh6Δ oye3Δ ari1Δ, P$_{TEF1}$-SpyCas9-T$_{CYC1}$, P$_{TEF1}$-CroCPR-T$_{PRM9}$, P$_{PGK1}$-CroCYB5-T$_{IDP1}$, P$_{TDH3}$-CroG8H-T$_{ADH1}$, P$_{TPI1}$-Cro8HGO-B-T$_{IDP1}$, P$_{TEF2}$-CroIO-T$_{CYC1}$, P$_{FBA1}$-CroCYPADH-T$_{CPS1}$, P$_{PGK1}$-Cro7DLGT-T$_{VPS13}$, P$_{TEF2}$-Cro7DLH-T$_{CYC1}$, P$_{TDH3}$-CroLAMT-T$_{ADH1}$, P$_{TPI1}$-CroSLS-T$_{IDP1}$, P$_{FBA1}$-CroSTR-T$_{PRM9}$	strictosidine (from nepetalactol and tryptamine feeding)	This study
MIA-BKV-3	MAT**a**, his3D1, leu2-3_112, ura3-52, trp1-289, atf1Δ oye2Δ adh6Δ oye3Δ ari1Δ, P$_{TEF1}$-SpyCas9-T$_{CYC1}$, P$_{TEF1}$-CroCPR-T$_{PRM9}$, P$_{PGK1}$-CroCYB5-T$_{IDP1}$, P$_{TDH3}$-CroG8H-T$_{ADH1}$, P$_{TEF2}$-CroIO-T$_{CYC1}$, P$_{FBA1}$-CroCYPADH-T$_{CPS1}$, P$_{PGK1}$-Cro7DLGT-T$_{VPS13}$, P$_{TEF2}$-Cro7DLH-T$_{CYC1}$, P$_{TDH3}$-CroLAMT-T$_{ADH1}$, P$_{TPI1}$-CroSLS-T$_{IDP1}$, P$_{FBA1}$-CroSTR-T$_{PRM9}$	strictosidine (from nepetalactol and tryptamine feeding)	This study
MIA-BKV-5	MATa, his3D1, leu2-3_112, ura3-52, trp1-289, atf1Δ oye2Δ adh6Δ oye3Δ ari1Δ, P$_{TEF1}$-SpyCas9-T$_{CYC1}$, P$_{TEF1}$-CroCPR-T$_{PRM9}$, P$_{PGK1}$-CroCYB5-T$_{IDP1}$, P$_{TDH3}$-CroG8H-T$_{ADH1}$, P$_{FBA1}$-NcaISY-T$_{CYC1}$, P$_{TEF1}$-NcaMLPLA-T$_{ADH1}$, P$_{TEF2}$-CroIO-T$_{CYC1}$, P$_{FBA1}$-CroCYPADH-T$_{CPS1}$, P$_{PGK1}$-Cro7DLGT-T$_{VPS13}$, P$_{TEF2}$-Cro7DLH-T$_{CYC1}$, P$_{TDH3}$-CroLAMT-T$_{ADH1}$, P$_{TPI1}$-CroSLS-T$_{IDP1}$, P$_{FBA1}$-CroSTR-T$_{PRM9}$	strictosidine (from 8-oxo-geranial and tryptamine feeding)	This study
MIA-BS-5	MAT**a**, his3D1, leu2-3_112, ura3-52, trp1-289, atf1Δ oye2Δ adh6Δ oye3Δ ari1Δ, P$_{TEF1}$-SpyCas9-T$_{CYC1}$, P$_{TEF1}$-CroCPR-T$_{PRM9}$, P$_{PGK1}$-CroCYB5-T$_{IDP1}$, P$_{TDH3}$-CroG8H-T$_{ADH1}$, P$_{TPI1}$-Cro8HGO-B-T$_{IDP1}$, P$_{FBA1}$-NcaISY-T$_{CYC1}$, P$_{TEF1}$-NcaMLPLA-T$_{ADH1}$, P$_{TEF2}$-CroIO-T$_{CYC1}$, P$_{FBA1}$-CroCYPADH-T$_{CPS1}$, P$_{PGK1}$-Cro7DLGT-T$_{VPS13}$, P$_{TEF2}$-Cro7DLH-T$_{CYC1}$, P$_{TDH3}$-CroLAMT-T$_{ADH1}$, P$_{TPI1}$-CroSLS-T$_{IDP1}$, P$_{FBA1}$-CroSTR-T$_{PRM9}$	strictosidine (from geraniol and tryptamine feeding)	This study
MIA-CA-1	MAT**a**, his3D1, leu2-3_112, ura3-52, trp1-289, atf1Δ oye2Δ adh6Δ oye3Δ ari1Δ, P$_{TEF1}$-SpyCas9-T$_{CYC1}$, P$_{TEF1}$-CroCPR-T$_{PRM9}$, P$_{PGK1}$-CroCYB5-T$_{IDP1}$, P$_{TDH3}$-CroG8H-T$_{ADH1}$, P$_{TPI1}$-Cro8HGO-B-T$_{IDP1}$, P$_{FBA1}$-CroISY-T$_{CPS1}$, P$_{TEF2}$-CroIO-T$_{CYC1}$, P$_{FBA1}$-CroCYPADH-T$_{CPS1}$, P$_{PGK1}$-Cro7DLGT-T$_{VPS13}$, P$_{TEF2}$-Cro7DLH-T$_{CYC1}$, P$_{TDH3}$-CroLAMT-T$_{ADH1}$, P$_{TPI1}$-CroSLS-T$_{IDP1}$, P$_{FBA1}$-CroSTR-T$_{PRM9}$, P$_{TEF1}$-CroSGD-T$_{ADH1}$, P$_{PGK1}$-CroHYS-T$_{CYC1}$	strictosidine (from geraniol and tryptamine feeding) due to inactive CroSGD	This study
MIA-CA-2	MAT**a**, his3D1, leu2-3_112, ura3-52, trp1-289, atf1Δ oye2Δ adh6Δ oye3Δ ari1Δ, P$_{TEF1}$-SpyCas9-T$_{CYC1}$, P$_{TEF1}$-CroCPR-T$_{PRM9}$, P$_{PGK1}$-CroCYB5-T$_{IDP1}$, P$_{TDH3}$-CroG8H-T$_{ADH1}$, P$_{TPI1}$-Cro8HGO-B-T$_{IDP1}$, P$_{FBA1}$-CroISY-T$_{CPS1}$, P$_{TEF2}$-CroIO-	tetrahydroalstonine (from geraniol and	This study

	T_{CYC1}, P_{FBA1}-CroCYPADH-T_{CPS1}, P_{PGK1}-Cro7DLGT-T_{VPS13}, P_{TEF2}-Cro7DLH-T_{CYC1}, P_{TDH3}-CroLAMT-T_{ADH1}, P_{TPI1}-CroSLS-T_{IDP1}, P_{FBA1}-CroSTR-T_{PRM9}, P_{TEF1}-RseSGD-T_{ADH1}, P_{PGK1}-CroHYS-T_{CYC1}	tryptamine feeding)	
MIA-CH-A2	MAT**a**, his3D1, leu2-3_112, ura3-52, trp1-289, atf1Δ oye2Δ adh6Δ oye3Δ ari1Δ, P_{TEF1}-SpyCas9-T_{CYC1}, P_{TEF1}-CroCPR-T_{PRM9}, P_{PGK1}-CroCYB5-T_{IDP1}, P_{TDH3}-CroG8H-T_{ADH1}, P_{PGK1}-Vmi8HGO-A -T_{ADH1}, P_{FBA1}-NcaISY-T_{CYC1}, P_{TEF1}-NcaMLPLA-T_{ADH1}, P_{TEF2}-CroIO-T_{CYC1}, P_{FBA1}-CroCYPADH-T_{CPS1}, P_{PGK1}-Cro7DLGT-T_{VPS13}, P_{TEF2}-Cro7DLH-T_{CYC1}, P_{TDH3}-CroLAMT-T_{ADH1}, P_{TPI1}-CroSLS-T_{IDP1}, P_{FBA1}-CroSTR-T_{PRM9}	strictosidine (from geraniol and tryptamine feeding)	This study
MIA-CL	MAT**a**, his3D1, leu2-3_112, ura3-52, trp1-289, atf1Δ oye2Δ adh6Δ oye3Δ ari1Δ, P_{HXT1}-ERG9-T_{ERG9}, P_{HXT3}-ERG20-T_{ERG20}, P_{TEF1}-SpyCas9-T_{CYC1}, P_{TEF1}-CroCPR-T_{PRM9}, P_{PGK1}-CroCYB5-T_{IDP1}, P_{TDH3}-CroG8H-T_{ADH1}, P_{PGK1}-Vmi8HGO-A -T_{ADH1}, P_{FBA1}-NcaISY-T_{CYC1}, P_{TEF1}-NcaMLPLA-T_{ADH1}, P_{TEF2}-CroIO-T_{CYC1}, P_{FBA1}-CroCYPADH-T_{CPS1}, P_{PGK1}-Cro7DLGT-T_{VPS13}, P_{TEF2}-Cro7DLH-T_{CYC1}, P_{TDH3}-CroLAMT-T_{ADH1}, P_{TPI1}-CroSLS-T_{IDP1}, P_{FBA1}-CroSTR-T_{PRM9}	strictosidine (from geraniol and tryptamine feeding)	This study
MIA-CM-3	MAT**a**, his3D1, leu2-3_112, ura3-52, trp1-289, atf1Δ oye2Δ adh6Δ oye3Δ ari1Δ, P_{HXT1}-ERG9-T_{ERG9}, P_{HXT3}-ERG20-T_{ERG20}, P_{TEF1}-SpyCas9-T_{CYC1}, P_{TEF1}-CroCPR-T_{PRM9}, P_{PGK1}-CroCYB5-T_{IDP1}, P_{MLS1}-AgrGPPS2-T_{VPS13}, P_{FBA1}-GgaFPS^{N144W}-T_{IDP1}, P_{PGK1}-IDI1-T_{PRM9}, P_{TDH3}-tHMG1-T_{ADH1}, P_{ICL1}-ERG20$^{F96W, N127W}$-tCroGES-T_{CYC1}, P_{PGK1}-CroTDC-T_{PRM9}, P_{TDH3}-CroG8H-T_{ADH1}, P_{PGK1}-Vmi8HGO-A -T_{ADH1}, P_{FBA1}-NcaISY-T_{CYC1}, P_{TEF1}-NcaMLPLA-T_{ADH1}, P_{TEF2}-CroIO-T_{CYC1}, P_{FBA1}-CroCYPADH-T_{CPS1}, P_{PGK1}-Cro7DLGT-T_{VPS13}, P_{TEF2}-Cro7DLH-T_{CYC1}, P_{TDH3}-CroLAMT-T_{ADH1}, P_{TPI1}-CroSLS-T_{IDP1}, P_{FBA1}-CroSTR-T_{PRM9}	*de novo* strictosidine	This study
MIA-CM-5	MAT**a**, his3D1, leu2-3_112, ura3-52, trp1-289, atf1Δ oye2Δ adh6Δ oye3Δ ari1Δ, P_{HXT1}-ERG9-T_{ERG9}, P_{HXT3}-ERG20-T_{ERG20}, P_{TEF1}-SpyCas9-T_{CYC1}, P_{TEF1}-CroCPR-T_{PRM9}, P_{PGK1}-CroCYB5-T_{IDP1}, P_{TEF2}-AgrGPPS2-T_{VPS13}, P_{FBA1}-GgaFPS^{N144W}-T_{IDP1}, P_{PGK1}-IDI1-T_{PRM9}, P_{TDH3}-tHMG1-T_{ADH1}, p_{CCW12}-ERG20$^{F96W, N127W}$-tCroGES-T_{CYC1}, P_{PGK1}-CroTDC-T_{PRM9}, P_{TDH3}-CroG8H-T_{ADH1}, P_{PGK1}-Vmi8HGO-A -T_{ADH1}, P_{FBA1}-NcaISY-T_{CYC1}, P_{TEF1}-NcaMLPLA-T_{ADH1}, P_{TEF2}-CroIO-T_{CYC1}, P_{FBA1}-CroCYPADH-T_{CPS1}, P_{PGK1}-Cro7DLGT-T_{VPS13}, P_{TEF2}-Cro7DLH-T_{CYC1}, P_{TDH3}-CroLAMT-T_{ADH1}, P_{TPI1}-CroSLS-T_{IDP1}, P_{FBA1}-CroSTR-T_{PRM9}	*de novo* strictosidine	This study
MIA-CR-A	MAT**a**, his3D1, leu2-3_112, ura3-52, trp1-289, atf1Δ oye2Δ adh6Δ oye3Δ ari1Δ, P_{HXT1}-ERG9-T_{ERG9}, P_{HXT3}-ERG20-T_{ERG20}, P_{TEF1}-SpyCas9-T_{CYC1}, P_{TEF1}-CroCPR-T_{PRM9}, P_{PGK1}-CroCYB5-T_{IDP1}, P_{MLS1}-AgrGPPS2-T_{VPS13}, P_{FBA1}-GgaFPS^{N144W}-T_{IDP1}, P_{PGK1}-IDI1-T_{PRM9}, P_{TDH3}-tHMG1-T_{ADH1},	*de novo* tabersonine	This study

	P_{ICL1}-ERG20$^{F96W, N127W}$-tCroGES-T_{CYC1}, P_{PGK1}-CroTDC-T_{PRM9}, P_{TDH3}-CroG8H-T_{ADH1}, P_{PGK1}-Vmi8HGO-A -T_{ADH1}, P_{FBA1}-NcaISY-T_{CYC1}, P_{TEF1}-NcaMLPLA-T_{ADH1}, P_{TEF2}-CroIO-T_{CYC1}, P_{FBA1}-CroCYPADH-T_{CPS1}, P_{PGK1}-Cro7DLGT-T_{VPS13}, P_{TEF2}-Cro7DLH-T_{CYC1}, P_{TDH3}-CroLAMT-T_{ADH1}, P_{TPI1}-CroSLS-T_{IDP1}, P_{FBA1}-CroSTR-T_{PRM9}, P_{TPI1}-RseSGD-T_{PRM9}, P_{PGK1}-CroGS-T_{IDP1}, P_{TEF2}-CroGO-T_{VPS13}, P_{TDH3}-CroRedox1-T_{CPS1}, P_{FBA1}-CroRedox2-T_{CYC1}, P_{TEF1}-CroSAT-T_{ADH1}, P_{PGK1}-CroPAS-T_{IDP1}, P_{TDH3}-CroDPAS-T_{PRM9}, P_{FBA1}-CroTS-T_{VPS13}		
MIA-CW-1	MAT**a**, his3D1, leu2-3_112, ura3-52, trp1-289, atf1Δ oye2Δ adh6Δ oye3Δ ari1Δ, P_{HXT1}-ERG9-T_{ERG9}, P_{HXT3}-ERG20-T_{ERG20}, P_{TEF1}-SpyCas9-T_{CYC1}, P_{TEF1}-CroCPR-T_{PRM9}, P_{PGK1}-CroCYB5-T_{IDP1}, P_{MLS1}-AgrGPPS2-T_{VPS13}, P_{FBA1}-GgaFPS^{N144W}-T_{IDP1}, P_{PGK1}-IDI1-T_{PRM9}, P_{TDH3}-tHMG1-T_{ADH1}, P_{ICL1}-ERG20$^{F96W, N127W}$-tCroGES-T_{CYC1}, P_{PGK1}-CroTDC-T_{PRM9}, P_{TDH3}-CroG8H-T_{ADH1}, P_{PGK1}-Vmi8HGO-A -T_{ADH1}, P_{FBA1}-NcaISY-T_{CYC1}, P_{TEF1}-NcaMLPLA-T_{ADH1}, P_{TEF2}-CroIO-T_{CYC1}, P_{FBA1}-CroCYPADH-T_{CPS1}, P_{PGK1}-Cro7DLGT-T_{VPS13}, P_{TEF2}-Cro7DLH-T_{CYC1}, P_{TDH3}-CroLAMT-T_{ADH1}, P_{TPI1}-CroSLS-T_{IDP1}, P_{FBA1}-CroSTR-T_{PRM9}, P_{TPI1}-RseSGD-T_{PRM9}, P_{PGK1}-CroGS-T_{IDP1}, P_{TEF2}-CroGO-T_{VPS13}, P_{TDH3}-CroRedox1-T_{CPS1}, P_{FBA1}-CroRedox2-T_{CYC1}, P_{TEF1}-CroSAT-T_{ADH1}, P_{PGK1}-CroPAS-T_{IDP1}, P_{TDH3}-CroDPAS-T_{PRM9}, P_{FBA1}-CroTS-T_{VPS13}, P_{TEF1}-CroT16H2-T_{ADH1}, P_{PGK1}-Cro16OMT-T_{IDP1}, P_{TDH3}-CroT3O-T_{PRM9}, P_{FBA1}-CroT3R-T_{VPS13}, P_{TEF1}-CroNMT-T_{ADH1}, P_{PGK1}-CroD4H-T_{IDP1}, P_{TDH3}-CroDAT-T_{PRM9}	*de novo* vindoline	This study
MIA-DA	MAT**a**, his3D1, leu2-3_112, ura3-52, trp1-289, P_{TEF1}-SpyCas9-T_{CYC1}, P_{TEF1}-CroCPR-T_{PRM9}, P_{PGK1}-CroCYB5-T_{VPS13},	no product	This study
MIA-DC	MAT**a**, his3D1, leu2-3_112, ura3-52, trp1-289, P_{TEF1}-SpyCas9-T_{CYC1}, P_{TEF1}-CroCPR-T_{PRM9}, P_{PGK1}-CroCYB5-T_{VPS13}, P_{TEF1}-CroSTR-T_{ADH1}, P_{TPI1}-RseSGD-T_{PRM9}, P_{PGK1}-CroGS-T_{IDP1}, P_{TEF2}-CroGO-T_{VPS13}, P_{TDH3}-CroRedox1-T_{CPS1}, P_{FBA1}-CroRedox2-T_{CYC1}, P_{TEF1}-CroSAT-T_{ADH1}, P_{PGK1}-CroPAS-T_{IDP1}, P_{TDH3}-CroDPAS-T_{PRM9}, P_{FBA1}-CroTS-T_{VPS13}, P_{TEF2}-CroCS-T_{CYC1}	tabersonine & catharanthine (from secologanin and tryptamine feeding)	This study
MIA-DE	MAT**a**, his3D1, leu2-3_112, ura3-52, trp1-289, P_{TEF1}-SpyCas9-T_{CYC1}, P_{TEF1}-CroCPR-T_{PRM9}, P_{PGK1}-CroCYB5-T_{VPS13}, P_{TEF2}-CroT16H1-T_{CPS1}, P_{TEF1}-CroT16H2-T_{ADH1}, P_{PGK1}-Cro16OMT-T_{IDP1}, P_{TDH3}-CroT3O-T_{PRM9}, P_{FBA1}-CroT3R-T_{VPS13}, P_{TEF1}-CroNMT-T_{ADH1}, P_{PGK1}-CroD4H-T_{IDP1}, P_{TDH3}-CroDAT-T_{PRM9}, P_{FBA1}-CroPRX1-T_{VPS13}	vindoline (from tabersonine feeding)	This study
MIA-DJ	MAT**a**, his3D1, leu2-3_112, ura3-52, trp1-289, P_{TEF1}-SpyCas9-T_{CYC1}, P_{TEF1}-CroCPR-T_{PRM9}, P_{PGK1}-CroCYB5-T_{VPS13}, P_{TEF1}-CroSTR-T_{ADH1}, P_{TPI1}-RseSGD-T_{PRM9}, P_{PGK1}-CroGS-T_{IDP1}, P_{TEF2}-CroGO-T_{VPS13}, P_{TDH3}-CroRedox1-T_{CPS1}, P_{FBA1}-CroRedox2-T_{CYC1}, P_{TEF1}-CroSAT-T_{ADH1}, P_{PGK1}-CroPAS-T_{IDP1}, P_{TDH3}-CroDPAS-T_{PRM9}, P_{FBA1}-CroTS-T_{VPS13}, P_{TEF2}-CroCS-	vindoline & catharanthine (from secologanin and tryptamine feeding)	This study

	T_{CYC1}, P_{TEF2}-CroT16H1-T_{CPS1}, P_{TEF1}-CroT16H2-T_{ADH1}, P_{PGK1}-Cro16OMT-T_{IDP1}, P_{TDH3}-CroT3O-T_{PRM9}, P_{FBA1}-CroT3R-T_{VPS13}, P_{TEF1}-CroNMT-T_{ADH1}, P_{PGK1}-CroD4H-T_{IDP1}, P_{TDH3}-CroDAT-T_{PRM9}, P_{FBA1}-CroPRX1-T_{VPS13}		
MIA-EM-1	MAT**a**, his3D1, leu2-3_112, ura3-52, trp1-289, atf1Δ oye2Δ adh6Δ oye3Δ ari1Δ, P_{HXT1}-ERG9-T_{ERG9}, P_{HXT3}-ERG20-T_{ERG20}, P_{TEF1}-SpyCas9-T_{CYC1}, P_{TEF1}-CroCPR-T_{PRM9}, P_{TEF1}-CroCPR- T_{ADH1}, P_{PGK1}-CroCYB5-T_{IDP1}, P_{MLS1}-AgrGPPS2-T_{VPS13}, P_{TEF2}-AgrGPPS2-T_{VPS13}, P_{FBA1}-GgaFPS^{N144W}-T_{IDP1}, P_{PGK1}-IDI1-T_{PRM9}, P_{TDH3}-tHMG1-T_{ADH1}, P_{ICL1}-ERG20$^{F96W, N127W}$-tCroGES-T_{CYC1}, P_{CCW12}-ERG20$^{F96W, N127W}$-tCroGES-$T_{PRM9,}$ P_{PGK1}-CroTDC-T_{PRM9}, P_{TDH3}-CroG8H-T_{ADH1}, P_{PGK1}-Vmi8HGO-A -T_{ADH1}, P_{FBA1}-NcaISY-T_{CYC1}, P_{TEF1}-NcaMLPLA-T_{ADH1}, P_{TEF2}-CroIO-T_{CYC1}, P_{FBA1}-CroCYPADH-T_{CPS1}, P_{PGK1}-Cro7DLGT-T_{VPS13}, P_{TEF2}-Cro7DLH-T_{CYC1}, P_{TDH3}-CroLAMT-T_{ADH1}, P_{TPI1}-CroSLS-T_{IDP1}, P_{FBA1}-CroSTR-T_{PRM9}, P_{TPI1}-RseSGD-T_{PRM9}, 2x P_{PGK1}-CroGS-T_{IDP1}, P_{TEF2}-CroGO-T_{VPS13}, P_{TDH3}-CroGO-T_{CPS1}, P_{TDH3}-CroRedox1-T_{CPS1}, P_{FBA1}-CroRedox2-T_{CYC1}, P_{TEF1}-CroSAT-T_{ADH1}, P_{PGK1}-CroPAS-T_{IDP1}, P_{TDH3}-CroDPAS-T_{PRM9}, P_{FBA1}-CroTS-T_{VPS13}, P_{TEF1}-CroT16H2-T_{ADH1}, P_{PGK1}-Cro16OMT-T_{IDP1}, P_{TDH3}-CroT3O-T_{PRM9}, P_{FBA1}-CroT3R-T_{VPS13}, P_{TEF1}-CroNMT-T_{ADH1}, P_{PGK1}-CroD4H-T_{IDP1}, P_{TDH3}-CroDAT-T_{PRM9}, P_{GAL1}-CroCS-T_{CYC1}	*de novo* vindoline & catharanthine	This study
MIA-EM-2	MAT**a**, his3D1, leu2-3_112, ura3-52, trp1-289, atf1Δ oye2Δ adh6Δ oye3Δ ari1Δ, P_{HXT1}-ERG9-T_{ERG9}, P_{HXT3}-ERG20-T_{ERG20}, P_{TEF1}-SpyCas9-T_{CYC1}, P_{TEF1}-CroCPR-T_{PRM9}, P_{TEF1}-CroCPR- T_{ADH1}, P_{PGK1}-CroCYB5-T_{IDP1}, P_{MLS1}-AgrGPPS2-T_{VPS13}, P_{TEF2}-AgrGPPS2-T_{VPS13}, P_{FBA1}-GgaFPS^{N144W}-T_{IDP1}, P_{PGK1}-IDI1-T_{PRM9}, P_{TDH3}-tHMG1-T_{ADH1}, P_{ICL1}-ERG20$^{F96W, N127W}$-tCroGES-T_{CYC1}, P_{CCW12}-ERG20$^{F96W, N127W}$-tCroGES-$T_{PRM9,}$ P_{PGK1}-CroTDC-T_{PRM9}, P_{TDH3}-CroG8H-T_{ADH1}, P_{PGK1}-Vmi8HGO-A -T_{ADH1}, P_{FBA1}-NcaISY-T_{CYC1}, P_{TEF1}-NcaMLPLA-T_{ADH1}, P_{TEF2}-CroIO-T_{CYC1}, P_{FBA1}-CroCYPADH-T_{CPS1}, P_{PGK1}-Cro7DLGT-T_{VPS13}, P_{TEF2}-Cro7DLH-T_{CYC1}, P_{TDH3}-CroLAMT-T_{ADH1}, P_{TPI1}-CroSLS-T_{IDP1}, P_{FBA1}-CroSTR-T_{PRM9}, P_{TPI1}-RseSGD-T_{PRM9}, 2x P_{PGK1}-CroGS-T_{IDP1}, P_{FBA1}-CroGS-T_{CYC1}, 2x P_{TEF2}-CroGO-T_{VPS13}, P_{TDH3}-CroGO-T_{CPS1}, P_{TDH3}-CroRedox1-T_{CPS1}, P_{FBA1}-CroRedox2-T_{CYC1}, 2x P_{TEF1}-CroSAT-T_{ADH1}, 2x P_{PGK1}-CroPAS-T_{IDP1}, P_{TDH3}-CroDPAS-T_{PRM9}, P_{TDH3}-CroDPAS-T_{CPS1}, P_{FBA1}-CroTS-T_{VPS13}, P_{CCW12}-CroTSnat- T_{PRM9},_P_{TEF1}-CroT16H2-T_{ADH1}, P_{PGK1}-Cro16OMT-T_{IDP1}, P_{TDH3}-CroT3O-T_{PRM9}, P_{FBA1}-CroT3R-T_{VPS13}, P_{TEF1}-CroNMT-T_{ADH1}, P_{PGK1}-CroD4H-T_{IDP1}, P_{TDH3}-CroDAT-T_{PRM9}, P_{GAL1}-CroCS-T_{CYC1}	de novo vindoline and catharanthine	This study
MIA-EM-3	MAT**a**, his3D1, leu2-3_112, ura3-52, trp1-289, atf1Δ oye2Δ adh6Δ oye3Δ ari1Δ, P_{HXT1}-ERG9-T_{ERG9}, P_{HXT3}-ERG20-T_{ERG20}, P_{TEF1}-SpyCas9-T_{CYC1}, P_{TEF1}-CroCPR-T_{PRM9}, P_{TEF1}-CroCPR- T_{ADH1}, P_{PGK1}-CroCYB5-T_{IDP1}, P_{MLS1}-AgrGPPS2-	de novo vindoline and catharanthine	This study

	T_{VPS13}, P_{TEF2}-AgrGPPS2-T_{VPS13}, P_{FBA1}-GgaFPS^{N144W}-T_{IDP1}, P_{PGK1}-IDI1-T_{PRM9}, P_{TDH3}-tHMG1-T_{ADH1}, P_{ICL1}-ERG20$^{F96W, N127W}$-tCroGES-T_{CYC1}, P_{CCW12}-ERG20$^{F96W, N127W}$-tCroGES-T_{PRM9}, P_{PGK1}-CroTDC-T_{PRM9}, P_{TDH3}-CroG8H-T_{ADH1}, P_{PGK1}-Vmi8HGO-A -T_{ADH1}, P_{FBA1}-NcaISY-T_{CYC1}, P_{TEF1}-NcaMLPLA-T_{ADH1}, P_{TEF2}-CroIO-T_{CYC1}, P_{FBA1}-CroCYPADH-T_{CPS1}, P_{PGK1}-Cro7DLGT-T_{VPS13}, P_{TEF2}-Cro7DLH-T_{CYC1}, P_{TDH3}-CroLAMT-T_{ADH1}, P_{TPI1}-CroSLS-T_{IDP1}, P_{FBA1}-CroSTR-T_{PRM9}, P_{TPI1}-RseSGD-T_{PRM9}, 2x P_{PGK1}-CroGS-T_{IDP1}, P_{TEF2}-CroGO-T_{VPS13}, P_{TDH3}-CroGO-T_{CPS1}, P_{TDH3}-CroRedox1-T_{CPS1}, P_{FBA1}-CroRedox2-T_{CYC1}, P_{TEF1}-CroSAT-T_{ADH1}, P_{PGK1}-CroPAS-T_{IDP1}, P_{TDH3}-CroDPAS-T_{PRM9}, P_{FBA1}-CroTS-T_{VPS13}, P_{CCW12}-CroTSnat-T_{CPS1}, 2x P_{TEF1}-CroT16H2-T_{ADH1}, P_{PGK1}-Cro16OMT-T_{IDP1}, 2x P_{TDH3}-CroT3O-T_{PRM9}, 2x P_{FBA1}-CroT3R-T_{VPS13}, P_{TEF1}-CroNMT-T_{ADH1}, 2x P_{PGK1}-CroD4H-T_{IDP1}, P_{TDH3}-CroDAT-T_{PRM9} , P_{TEF2}-CroDAT-T_{CYC1}, P_{GAL1}-CroCS-T_{CYC1}		
MIA-GF-01	MAT**a**, his3D1, leu2-3_112, ura3-52, trp1-289, P_{TEF1}-SpyCas9-T_{CYC1}, P_{TDH3}-CroGS-yEGFP-T_{ADH1}	Red marker in the nucleus	This study
MIA-GF-02	MAT**a**, his3D1, leu2-3_112, ura3-52, trp1-289, P_{TEF1}-SpyCas9-T_{CYC1}, P_{TDH3}-CroGO-yEGFP-T_{ADH1}	Red marker on the ER membrane	This study
MIA-GF-03	MAT**a**, his3D1, leu2-3_112, ura3-52, trp1-289, P_{TEF1}-SpyCas9-T_{CYC1}, P_{TDH3}-CroRedox1-yEGFP-T_{ADH1}	Red marker in the nucleus	This study
MIA-GF-04	MAT**a**, his3D1, leu2-3_112, ura3-52, trp1-289, P_{TEF1}-SpyCas9-T_{CYC1}, P_{TDH3}-CroRedox2-yEGFP-T_{ADH1}	Red marker in the nucleus	This study
MIA-GF-05	MAT**a**, his3D1, leu2-3_112, ura3-52, trp1-289, P_{TEF1}-SpyCas9-T_{CYC1}, P_{TDH3}-CroSAT-yEGFP-T_{ADH1}	Red marker in the nucleus	This study
MIA-GF-06	MAT**a**, his3D1, leu2-3_112, ura3-52, trp1-289, P_{TEF1}-SpyCas9-T_{CYC1}, P_{TDH3}-CroPAS-yEGFP-T_{ADH1}	Red marker in the nucleus	This study
MIA-GF-07	MAT**a**, his3D1, leu2-3_112, ura3-52, trp1-289, P_{TEF1}-SpyCas9-T_{CYC1}, P_{TDH3}-CroDPAS-yEGFP-T_{ADH1}	Red marker in the nucleus	This study
MIA-GF-08	MAT**a**, his3D1, leu2-3_112, ura3-52, trp1-289, P_{TEF1}-SpyCas9-T_{CYC1}, P_{TDH3}-CroTS-yEGFP-T_{ADH1}	Red marker in the nucleus	This study
MIA-GF-09	MAT**a**, his3D1, leu2-3_112, ura3-52, trp1-289, P_{TEF1}-SpyCas9-T_{CYC1}, P_{TDH3}-CroCS-yEGFP-T_{ADH1}	Red marker in the nucleus	This study
MIA-GF-10-A	MAT**a**, his3D1, leu2-3_112, ura3-52, trp1-289, P_{TEF1}-SpyCas9-T_{CYC1}, P_{TDH3}-CroPRX1-yEGFP-T_{ADH1}	Red marker in the nucleus	This study
MIA-GF-10-B	MAT**a**, his3D1, leu2-3_112, ura3-52, trp1-289, P_{TEF1}-SpyCas9-T_{CYC1}, P_{TDH3}-tCroPRX1$_{N21\Delta}$-yEGFP-T_{ADH1}	Red marker in the mitochondria	This study
MIA-GF-10-C	MAT**a**, his3D1, leu2-3_112, ura3-52, trp1-289, P_{TEF1}-SpyCas9-T_{CYC1}, P_{TDH3}-tCroPRX1$_{N34\Delta}$-yEGFP-T_{ADH1}	Red marker in the nucleus	This study
MIA-GF-10-D	MAT**a**, his3D1, leu2-3_112, ura3-52, trp1-289, P_{TEF1}-SpyCas9-T_{CYC1}, P_{TDH3}-tCroPRX1$_{C25\Delta}$-yEGFP-T_{ADH1}	Red marker in the nucleus	This study

MIA-GF-10-E	MAT**a**, his3D1, leu2-3_112, ura3-52, trp1-289, P_{TEF1}-SpyCas9-T_{CYC1}, P_{TDH3}-tCroPRX1$_{N21\Delta C25\Delta}$-yEGFP-T_{ADH1}	Red marker in the mitochondria	This study
MIA-GF-10-F	MAT**a**, his3D1, leu2-3_112, ura3-52, trp1-289, P_{TEF1}-SpyCas9-T_{CYC1}, P_{TDH3}-tCroPRX1$_{N34\Delta C25\Delta}$-yEGFP-T_{ADH1}	Red marker in the mitochondria	This study
MIA-GF-17	MAT**a**, his3D1, leu2-3_112, ura3-52, trp1-289, P_{TEF1}-SpyCas9-T_{CYC1}, $P_{TDH3EF1}$-CroG8H-yEGFP-T_{ADH1}	Red marker on the ER membrane	This study
MIA-GF-18	MAT**a**, his3D1, leu2-3_112, ura3-52, trp1-289, P_{TEF1}-SpyCas9-T_{CYC1}, $P_{TEF1}P_{TDH3}$-CroIO-yEGFP-T_{ADH1}	Red marker on the ER membrane	This study
MIA-GF-19	MAT**a**, his3D1, leu2-3_112, ura3-52, trp1-289, P_{TEF1}-SpyCas9-T_{CYC1}, $P_{TEF1}P_{TDH3}$-Cro7DLH-yEGFP-T_{ADH1}	Red marker on the ER membrane	This study
MIA-GF-20	MAT**a**, his3D1, leu2-3_112, ura3-52, trp1-289, P_{TEF1}-SpyCas9-T_{CYC1}, $P_{TEF1}P_{TDH3}$-CroSLS-yEGFP-T_{ADH1}	Red marker on the ER membrane	This study
MIA-GF-21-A	MAT**a**, his3D1, leu2-3_112, ura3-52, trp1-289, P_{TEF1}-SpyCas9-T_{CYC1}, $P_{TEF1}P_{TDH3}$-tCroSTR-yEGFP-T_{ADH1}	Red marker in the nucleus	This study
MIA-GF-21-B	MAT**a**, his3D1, leu2-3_112, ura3-52, trp1-289, P_{TEF1}-SpyCas9-T_{CYC1}, $P_{TEF1}P_{TDH3}$-CroSTR-yEGFP-T_{ADH1}	Red marker in the vacuole	This study
MIA-GF-22-A	MAT**a**, his3D1, leu2-3_112, ura3-52, trp1-289, P_{TEF1}-SpyCas9-T_{CYC1}, $P_{TEF1}P_{TDH3}$-yEGFP-RseSGD-T_{ADH1}	Red marker in the nucleus	This study
MIA-GF-22-B	MAT**a**, his3D1, leu2-3_112, ura3-52, trp1-289, P_{TEF1}-SpyCas9-T_{CYC1}, $P_{TEF1}P_{TDH3}$- yEGFP-RseCroSGD-T_{ADH1}	Red marker in the nucleus	This study
MIA-GF-22-C	MAT**a**, his3D1, leu2-3_112, ura3-52, trp1-289, P_{TEF1}-SpyCas9-T_{CYC1}, $P_{TEF1}P_{TDH3}$-yEGFP-hybrid SGD$_{C1C2R3R4}$-T_{ADH1}	Red marker in the nucleus	This study
MIA-GF-22-D	MAT**a**, his3D1, leu2-3_112, ura3-52, trp1-289, P_{TEF1}-SpyCas9-T_{CYC1}, $P_{TEF1}P_{TDH3}$-yEGFP-hybrid SGD$_{C1C2R3C4}$-T_{ADH1}	Red marker in the nucleus	This study

Supplementary Table 3. Protein sequences of four domains from *Rse*SGD and *Cro*SGD

Domain	Short name	Amino acid sequence
*Cro*SGD domain 1 (*Cro*SGD-D_1)	C_1	MGSKDDQSLVVAISPAAEPNGNHSVPIPFAYPSIPIQPRKHNKPIVHRRDFPSDFILGAGGSAYQCEGAYNEGNRGPSIWDTFTNRYPAKIADGSNGNQAINSYNLYKEDIKIMKQTGLESYRFSI
*Cro*SGD domain 2 (*Cro*SGD-D_2)	C_2	SWSRVLPGGNLSGGVNKDGVKFYHDFIDELLANGIKPFATLFHWDLPQALEDEYGGFLSDRIVEDFTEYAEFCFWEFGDKVKFWTTFNEPHTYVASGYATGEFAPGRGGADGKGNPGKEPYIATHNLLLSHKAAVEVYRKNFQKCQGGEIGI
*Cro*SGD domain 3 (*Cro*SGD-D_3)	C_3	VLNSMWMEPLNETKEDIDARERGPDFMLGWFIEPLTTGEYPKSMRALVGSRLPEFSTEDSEKLTGCYDFIGMNYYTTTYVSNADKIPDTPGYETDARINKNIFVKKVDGKEVRIGEPCYGGWQHVVPSGLYNLLVYTKEKYHVPVIYVSECGVVEENRTNILLTEGKTNILLTEARHDKLRVDFLQSHLASVRDAIDDGV
*Cro*SGD domain 4 (*Cro*SGD-D_4)	C_4	NVKGFFVWSFFDNFEWNLGYICRYGIIHVDYKTFQRYPKDSAIWYKNFISEGFVTNTAKKRFREEDKLVELVKKQKY
*Rse*SGD domain 1 (*Rse*SGD-D_1)	R_1	MDNTQAEPLVVAIVPKPNASTEHTNSHLIPVTRSKIVVHRRDFPQDFIFGAGGSAYQCEGAYNEGNRGPSIWDTFTQRSPAKISDGSNGNQAINCYHMYKEDIKIMKQTGLESYRFSI
*Rse*SGD domain 2 (*Rse*SGD-D_2)	R_2	SWSRVLPGGRLAAGVNKDGVKFYHDFIDELLANGIKPSVTLFHWDLPQALEDEYGGFLSHRIVDDFCEYAEFCFWEFGDKIKYWTTFNEPHTFAVNGYALGEFAPGRGGKGDEGDPAIEPYVVTHNILLAHKAAVEEYRNKFQKCQEGEIGI
*Rse*SGD domain 3 (*Rse*SGD-D_3)	R_3	VLNSMWMEPLSDVQADIDAQKRALDFMLGWFLEPLTTGDYPKSMRELVKGRLPKFSADDSEKLKGCYDFIGMNYYTATYVTNAVKSNSEKLSYETDDQVTKTFERNQKPIGHALYGGWQHVVPWGLYKLLVYTKETYHVPVLYVTESGMVEENKTKILLSEARRDAERTDYHQKHLASVRDAIDDGV
*Rse*SGD domain 4 (*Rse*SGD-D_4)	R_4	NVKGYFVWSFFDNFEWNLGYICRYGIIHVDYKSFERYPKESAIWYKNFIAGKSTTSPAKRRREEAQVELVKRQKT

Supplementary Table 4. List of plasmids

ID	Description	Reference
pCfB-54	2 µ, pESC-URA3 USER vector	Jensen et al. [5]
pCfB-55	2 µ, pESC-HIS3 USER vector	Jensen et al. [5]
pCfB-56	2 µ, pESC-LEU2 USER vector	Jensen et al. [5]
pCfB-57	2 µ, pESC-TRP1 USER vector	Jensen et al. [5]
pCfB-1767	CEN6/ARS4, pRS414-TRP1, P_{TEF1}-SpyCas9-T_{CYC1}	
pCfB-2480	CEN6/ARS4, pRS413-HIS3 USER vector 1 (pRS413U)	This study
pCfB-4054	CEN6/ARS4, pRS413-HIS3 USER vector 2 (pRS413U_2)	This study
pCfB-4055	CEN6/ARS4, pRS413-HIS3 USER vector 3 (pRS413U_3)	This study
pCfB-9566	CEN6/ARS4, pRS413-HIS3 USER vector 4 (pRS413U_4)	This study
pCfB-4084	CEN6/ARS4, pRS413-HIS3 USER vector 5 (pRS413U_5)	This study
pCfB-4085	CEN6/ARS4, pRS413-HIS3 USER vector 6 (pRS413U_6)	This study
pCfB-9551	CEN6/ARS4, pRS413-HIS3 USER vector 7 (pRS413U_7)	This study
pCfB-2481	CEN6/ARS4, pRS414-TRP1 USER vector (pRS414U)	This study
pCfB-2482	CEN6/ARS4, pRS415-LEU2 USER vector (pRS415U)	This study
pCfB-2483	CEN6/ARS4, pRS416-URA3 USER vector (pRS416U)	This study
pCfB-4063	2 µ, pESC-LEU2, gRNA-ATF1	This study
pCfB-4065	2 µ, pESC-LEU2, gRNA-OYE2	This study
pCfB-4092	2 µ, pESC-LEU2, 2xgRNA-[ATF1, OYE2]	This study
pCfB-9497	2 µ, pESC-URA3, gRNA-ADH6-1	This study
pCfB-9498	2 µ, pESC-URA3, gRNA-OYE3-1	This study
pCfB-9499	2 µ, pESC-URA3, gRNA-OYE3-2	This study
pCfB-9500	2 µ, pESC-URA3, gRNA-ARI1-1	This study
pCfB-9501	2 µ, pESC-URA3, gRNA-ARI1-2	This study
pCfB-9503	2 µ, pESC-LEU2, 4xgRNA-[OYE3-1, OYE3-2, ARI1, ADH6]	This study
pCfB-9657	2 µ, pESC-LEU2, 2xgRNA-[P_{ERG20}, P_{ERG9}]	This study
pCfB-9649	2 µ, pESC-URA3, gRNA-HMRa-3	This study
pCfB-6910	2 µ, pESC-URA3, gRNA-X2	This study
pCfB-6911	2 µ, pESC-URA3, gRNA-X3	This study
pCfB-6912	2 µ, pESC-URA3, gRNA-X4	This study
pCfB-6913	2 µ, pESC-URA3, gRNA-XI1	This study
pCfB-6914	2 µ, pESC-URA3, gRNA-XI2	This study
pCfB-6915	2 µ, pESC-URA3, gRNA-XI3	This study
pCfB-6916	2 µ, pESC-URA3, gRNA-XI5	This study
pCfB-6917	2 µ, pESC-URA3, gRNA-XII1	This study
pCfB-6918	2 µ, pESC-URA3, gRNA-XII2	This study

pCfB-6919	2 µ, pESC-URA3, gRNA-XII4	This study
pCfB-6920	2 µ, pESC-URA3, gRNA-XII5	This study
pCfB-9543	2 µ, pESC-URA3, gRNA-Syn1	This study
pCfB-9544	2 µ, pESC-URA3, gRNA-Syn2	This study
pCfB-9634	2 µ, pESC-URA3, gRNA-X2b	This study
pCfB-9635	2 µ, pESC-URA3, gRNA-XI2b	This study
pCfB-9636	2 µ, pESC-URA3, gRNA-XI5b	This study
pCfB-9637	2 µ, pESC-URA3, gRNA-XII1b	This study
pCfB-9638	2 µ, pESC-URA3, gRNA-XII5b	This study
pCfB-9658	2 µ, pESC-LEU2, gRNA-IV1-1	This study
pCfB-9661	2 µ, pESC-LEU2, gRNA-IV2-2	This study
pCfB-7768	CEN6/ARS4, pRS414U, P_{TEF1}-CroG8H-yEGFP-T_{ADH1}	This study
pCfB-7788	CEN6/ARS4, pRS413U, P_{TEF1}-CroSLS-yEGFP-T_{ADH1}	This study
pCfB-7789	CEN6/ARS4, pRS413U, P_{TEF1}-CroIO-yEGFP-T_{ADH1}	This study
pCfB-7790	CEN6/ARS4, pRS413U, P_{TEF1}-Cro7DLH-yEGFP-T_{ADH1}	This study
pCfB-4676	CEN6/ARS4, pRS416U, P_{TDH3}-CroG8H-T_{ADH1}, P_{TEF1}-CroSLS-T_{CYC1}	This study
pCfB-4069	CEN6/ARS4, pRS413U_3, P_{TEF1}-tCroSTR-T_{PRM9}, P_{PGK1}-tCroGES-T_{VPS13}	This study
pCfB-4070	CEN6/ARS4, pRS413U_2, P_{TDH3}-Cro7DLH-T_{ADH1}, P_{FBA1}-Cro8HGO-T_{IDP1}	This study
pCfB-4071	CEN6/ARS4, pRS413U_3, P_{PGK1}-CroTDC-T_{PRM9}, P_{TEF1}-AgrGPPS2-T_{VPS13}	This study
pCfB-4072	CEN6/ARS4, pRS413U_2, P_{TDH3}-CroLAMT-T_{ADH1}, P_{FBA1}-GgaFPS144-T_{IDP1}	This study
pCfB-4073	CEN6/ARS4, pRS413U_3, P_{TEF1}-CroIO-T_{PRM9}, P_{PGK1}-Cro7DLGT-T_{VPS13}	This study
pCfB-4074	CEN6/ARS4, pRS413U_2, P_{TDH3}-tHMG1-T_{ADH1}, P_{FBA1}-CroISY-T_{IDP1}	This study
pCfB-5384	CEN6/ARS4, pRS413U_3, P_{PGK1}-CroTDC-T_{PRM9}, P_{TEF1}-IDI1-T_{VPS13}	This study
pCfB-5385	CEN6/ARS4, pRS413U_2, P_{TDH3}-CroLAMT-T_{ADH1}, P_{FBA1}-CroCYPADH-T_{IDP1}	This study
pCfB-6883	CEN6/ARS4, pRS413U_2, P_{TDH3}-CroG8H-T_{ADH1}, P_{FBA1}-CroSLS-T_{IDP1}	This study
pCfB-6884	CEN6/ARS4, pRS413U_3, P_{TEF1}-CroCPR-T_{PRM9}, P_{PGK1}-CroCYB5-T_{VPS13}	This study
pCfB-11432	2 µ, pESC-URA3, P_{TEF1}-CroG8H-T_{ADH1}	This study
pCfB-11433	2 µ, pESC-URA3, P_{TEF1}-Cro8HGO-B-T_{ADH1}	This study
pCfB-11435	2 µ, pESC-URA3, P_{TEF1}-CroISY-T_{ADH1}	This study
pCfB-11436	2 µ, pESC-URA3, P_{TEF1}-CroIO-T_{ADH1}	This study
pCfB-11437	2 µ, pESC-URA3, P_{TEF1}-CroCYPADH-T_{ADH1}	This study
pCfB-11438	2 µ, pESC-URA3, P_{TEF1}-Cro7DLH-T_{ADH1}	This study
pCfB-11439	2 µ, pESC-URA3, P_{TEF1}-Cro7DLGT-T_{ADH1}	This study
pCfB-11440	2 µ, pESC-URA3, P_{TEF1}-CroLAMT-T_{ADH1}	This study
pCfB-11441	2 µ, pESC-URA3, P_{TEF1}-CroSLS-T_{ADH1}	This study
pCfB-11442	2 µ, pESC-URA3, P_{TEF1}-tCroSTR-T_{ADH1}	This study
pCfB-11443	2 µ, pESC-URA3, P_{TEF1}-CroCPR-T_{ADH1}	This study

pCfB-11444	2 µ, pESC-URA3, P_{TEF1}-CroCYB5-T_{ADH1}	This study
pCfB-9477	2 µ, pESC-HIS3, P_{TEF1}-CroLAMT-yEGFP-T_{ADH1}	This study
pCfB-9478	2 µ, pESC-HIS3, P_{TEF1}-CroG8H-yEGFP-T_{ADH1}	This study
pCfB-9479	2 µ, pESC-HIS3, P_{TEF1}-CroIO-yEGFP-T_{ADH1}	This study
pCfB-9480	2 µ, pESC-HIS3, P_{TEF1}-Cro7DLH-yEGFP-T_{ADH1}	This study
pCfB-9481	2 µ, pESC-HIS3,P_{TEF1}-CroSLS-yEGFP-T_{ADH1}	This study
pCfB-9482	2 µ, pESC-HIS3, P_{TEF1}-CroCPR-yEGFP-T_{ADH1}	This study
pCfB-9483	2 µ, pESC-HIS3, P_{PGK1}-CroTDC-yEGFP-T_{ADH1}	This study
pCfB-9484	2 µ, pESC-HIS3, P_{TEF1}-tCroSTR-yEGFP-T_{ADH1}	This study
pCfB-9485	2 µ, pESC-HIS3, P_{TEF1}-CroSGD-yEGFP-T_{ADH1}	This study
pCfB-9486	2 µ, pESC-HIS3, P_{TEF1}-6xHis-tCroSTR-yEGFP-T_{ADH1}	This study
pCfB-9487	2 µ, pESC-HIS3, P_{TEF1}-6xHis-tCroSTR-MBP-T_{ADH1}	This study
pCfB-9512	2 µ, pESC-HIS3, P_{TEF1}-CroSTR-T_{ADH1}	This study
pCfB-9513	2 µ, pESC-HIS3, P_{TEF1}-tCroSTR-T_{ADH1}	This study
pCfB-9514	2 µ, pESC-HIS3, P_{TEF1}-RspSTR1-T_{ADH1}	This study
pCfB-9515	2 µ, pESC-HIS3, P_{TEF1}-tRspSTR1-T_{ADH1}	This study
pCfB-9516	2 µ, pESC-HIS3, P_{TEF1}-OpuSTR-T_{ADH1}	This study
pCfB-9517	2 µ, pESC-HIS3, P_{TEF1}-tOpuSTR-T_{ADH1}	This study
pCfB-9518	2 µ, pESC-HIS3, P_{TEF1}-MnoSTR-T_{ADH1}	This study
pCfB-9519	2 µ, pESC-HIS3, P_{TEF1}-tMnoSTR-T_{ADH1}	This study
pCfB-9520	2 µ, pESC-HIS3, P_{TEF1}-AthSTR-T_{ADH1}	This study
pCfB-9521	2 µ, pESC-HIS3, P_{TEF1}-tAthSTR-T_{ADH1}	This study
pCfB-9522	2 µ, pESC-HIS3, P_{TEF1}-CacSTR-T_{ADH1}	This study
pCfB-9523	2 µ, pESC-HIS3, P_{TEF1}-tCacSTR-T_{ADH1}	This study
pCfB-9524	2 µ, pESC-HIS3, P_{TEF1}-TelSTR-T_{ADH1}	This study
pCfB-9525	2 µ, pESC-HIS3, P_{TEF1}-tTelSTR-T_{ADH1}	This study
pCfB-9526	2 µ, pESC-HIS3, P_{TEF1}-NmuNEPS2-T_{ADH1}	This study
pCfB-9527	CEN6/ARS4, pRS415U, P_{TEF1}-CroISY-GS-NmuNEPS2-T_{ADH1}	This study
pCfB-9528	CEN6/ARS4, pRS415U, P_{TEF1}-NmuNEPS2-linker-CroISY-T_{ADH1}	This study
pCfB-9533	CEN6/ARS4, pRS415U, P_{TEF1}- ERG20$^{F96W,\ N127W}$-T_{ADH1}, P_{PGK1}-tCroGES-T_{CYC1}	This study
pCfB-6845	CEN6/ARS4, pRS415U, P_{TEF1}- ERG20$^{F96W,\ N127W}$-tCroGES-T_{CYC1}	This study
pCfB-9859	CEN6/ARS4, pRS415U, P_{TEF1}-tCroGES-ERG20$^{F96W,\ N127W}$-T_{CYC1}	This study
pCfB-9534	CEN6/ARS4, pRS415U, P_{TEF1}-NmuNEPS2, P_{PGK1}-CroISY-T_{ADH1}	This study
pCfB-9529	CEN6/ARS4, pRS415U, P_{TEF1}-CroSGD-GS-CroTHAS-T_{CYC1}	This study
pCfB-9530	CEN6/ARS4, pRS415U, P_{TEF1}-CroTHAS-GS-CroSGD-T_{CYC1}	This study
pCfB-9531	CEN6/ARS4, pRS415U, P_{TEF1}-tCroSGD-GS-tCroTHAS-T_{CYC1}	This study
pCfB-9532	CEN6/ARS4, pRS415U, P_{TEF1}-tCroTHAS-GS-tCroSGD-T_{CYC1}	This study

pCfB-9535	CEN6/ARS4, pRS415U, P_{TEF1}-CroSGD-T_{ADH1}, P_{PGK1}-CroTHAS-T_{CYC1}	This study
pCfB-9536	CEN6/ARS4, pRS415U, P_{TEF1}-tCroSGD-T_{ADH1}, P_{PGK1}-tCroTHAS-T_{CYC1}	This study
pCfB-9547	CEN6/ARS4, pRS415U, P_{TEF1}-CroSGD-T_{ADH1}, P_{PGK1}-tCroTHAS-T_{CYC1}	This study
pCfB-9548	CEN6/ARS4, pRS415U, P_{TEF1}-tCroSGD-T_{ADH1}, P_{PGK1}-tCroTHAS-T_{CYC1}	This study
pCfB-9549	CEN6/ARS4, pRS415U, P_{TEF1}-RseSGD-T_{ADH1}, P_{PGK1}-CroTHAS-T_{CYC1}	This study
pCfB-9552	CEN6/ARS4, pRS413U_5, P_{TEF1}-CroSTR-T_{ADH1}, P_{PGK1}-CroGS-T_{IDP1}	This study
pCfB-9553	CEN6/ARS4, pRS413U_7, P_{CCW12}-RseSGD-T_{PRM9}, P_{TEF2}-CroGO-T_{VPS13}	This study
pCfB-9554	CEN6/ARS4, pRS413U_4, P_{TDH3}-CroRedox1-T_{CPS1}, P_{FBA1}-CroRedox2-T_{CYC1}	This study
pCfB-9555	CEN6/ARS4, pRS413U_5, P_{TEF1}-CroSAT-T_{ADH1}, P_{PGK1}-CroPAS-T_{IDP1}	This study
pCfB-9556	CEN6/ARS4, pRS413U_7, P_{TDH3}-CroDPAS-T_{PRM9}, P_{FBA1}-CroTS-T_{VPS13}	This study
pCfB-9557	CEN6/ARS4, pRS413U_4, P_{TEF2}-CroCS-T_{CYC1}	This study
pCfB-9558	CEN6/ARS4, pRS413U_5, P_{TEF1}-CroT16H2-T_{ADH1}, P_{PGK1}-Cro16OMT-T_{IDP1}	This study
pCfB-9559	CEN6/ARS4, pRS413U_6, P_{TDH3}-CroT3O-T_{PRM9}, P_{FBA1}-CroT3R-T_{VPS13}	This study
pCfB-9560	CEN6/ARS4, pRS413U_5, P_{TEF1}-CroNMT-T_{ADH1}, P_{PGK1}-CroD4H-T_{IDP1}	This study
pCfB-9561	CEN6/ARS4, pRS413U_7, P_{TDH3}-CroDAT-T_{PRM9}, P_{FBA1}-CroPRX1-T_{VPS13}	This study
pCfB-9562	CEN6/ARS4, pRS413U_4, P_{TEF2}-CroT16H1-T_{CYC1}	This study
pCfB-9565	CEN6/ARS4, pRS415U, P_{TEF1}-RseSGD-T_{ADH1}, P_{PGK1}-CroHYS-T_{CYC1}	This study
pCfB-9568	CEN6/ARS4, pRS415U, P_{TEF1}-CroSGD-T_{ADH1}	This study
pCfB-9569	CEN6/ARS4, pRS415U, P_{TEF1}-RseSGD-T_{ADH1}	This study
pCfB-9570	CEN6/ARS4, pRS415U, P_{TEF1}-RveSGD-T_{ADH1}	This study
pCfB-9571	CEN6/ARS4, pRS415U, P_{TEF1}-GseSGD-T_{ADH1}	This study
pCfB-9572	CEN6/ARS4, pRS415U, P_{TEF1}-CacSGD-T_{ADH1}	This study
pCfB-9573	CEN6/ARS4, pRS415U, P_{TEF1}-SapSGD-T_{ADH1}	This study
pCfB-9574	CEN6/ARS4, pRS415U, P_{TEF1}-UtoSGD-T_{ADH1}	This study
pCfB-9575	CEN6/ARS4, pRS415U, P_{TEF1}-GsoSGD-T_{ADH1}	This study
pCfB-10713	CEN6/ARS4, pRS-TRP1, P_{GAL10}-Vmi8HGO-A-T_{ADH1}	This study
pCfB-10715	CEN6/ARS4, pRS-TRP1, P_{GAL10}-Cro8HGO-A-T_{ADH1}	This study
pCfB-10716	CEN6/ARS4, pRS-HIS3, P_{GAL1}-Cro8HGO-B-T_{CYC1}	This study
pCfB-10717	CEN6/ARS4, pRS-HIS3, P_{GAL1}-Ptr8HGO-B-T_{CYC1}	This study
pCfB-10718	CEN6/ARS4, pRS-HIS3, P_{GAL1}-Rte8HGO-B-T_{CYC1}	This study
pCfB-10719	CEN6/ARS4, pRS-HIS3, P_{GAL1}-Sin8HGO-B-T_{CYC1}	This study
pCfB-10720	CEN6/ARS4, pRS-HIS3, P_{GAL1}-NmuISY-T_{CYC1}	This study
pCfB-10721	CEN6/ARS4, pRS-HIS3, P_{GAL1}-NcaISY-T_{CYC1}	This study
pCfB-10722	CEN6/ARS4, pRS-HIS3, P_{GAL1}-DpuISY-T_{CYC1}	This study
pCfB-10723	CEN6/ARS4, pRS-HIS3, P_{GAL10}-CroISY-T_{CYC1}	This study
pCfB-10724	CEN6/ARS4, pRS-HIS3, P_{GAL10}-OeuISY-T_{CYC1}	This study
pCfB-10725	CEN6/ARS4, pRS-LEU2, P_{GAL10}-NcaMLPLA-T_{ADH1}	This study

pCfB-10726	CEN6/ARS4, pRS-LEU2, P_{GAL10}-NcaMLPLB-T_{ADH1}	This study
pCfB-10727	CEN6/ARS4, pRS-LEU2, P_{GAL10}-NmuMLPL-T_{ADH1}	This study
pCfB-10728	CEN6/ARS4, pRS-LEU2, P_{GAL10}-NmuNEPS2-T_{ADH1}	This study
ZJOM90 (Addgene ID-133647)	Integration of SS-mCherry-HDEL in yeast genome using auxotrophic marker TRP1 from S. cerevisiae. Marker for yeast endoplasmic reticulum (ER).	Zhu et al.[6]
ZJOM82 (Addgene ID-133648)	Integration of NAB2-mCherry in yeast genome using auxotrophic marker TRP1 from Kluyveromyces lactis. Marker for yeast nucleus.	Zhu et al.[6]
ZJOM65 (Addgene ID-133654)	Integration of VPH1-mCherry in yeast genome using auxotrophic marker TRP1 from Kluyveromyces lactis. Marker for yeast vacuole.	Zhu et al.[6]
ZJOM64 (Addgene ID-133655)	Integration of COX4-DuDre in yeast genome using auxotrophic marker TRP1 from Kluyveromyces lactis. Marker for yeast mitochondria.	Zhu et al.[6]

Supplementary Table 5. List of chemicals for feeding and analytical standards

Chemical	CAS #	Supplier	Catolog #
nerol (internal standard)	106-25-2	Sigma-Aldrich	W277002
caffeine (internal standard)	58-08-2	Sigma-Aldrich	C0750
geraniol	106-24-1	Sigma-Aldrich	163333
8-hydroxygeraniol	26488-97-1	Toronto Research Chemicals (Canada)	H939740
8-oxogeranial	80054-40-6	Toronto Research Chemicals (Canada)	D476180
cis-trans-nepetalactol	109215-55-6	Toronto Research Chemicals (Canada)	N390065
citronellol	7540-51-4	Sigma-Aldrich	303488
loganic acid	22255-40-9	Carl Roth GmbH + Co (Germany)	4158.2
loganin	18524-94-2	Santa Cruz Biotechnology (USA)	sc-202696
secologanin	19351-63-4	Sigma-Aldrich	50741
tryptamine	61-54-1	Sigma-Aldrich	193747
strictosidine	20824-29-7	PHYTOCONSULT (Netherlands)	*discontinued*
tetrahydroalstonine	6474-90-4	Chengdu Push Bio-technology Co., Ltd. (China)	PS15041702
tabersonine	29479-00-3	Gift from Axyntis Group, France	N.A.
catharanthine	2468-21-5	Bionordika (Germany)	CC-11695
vindoline	2182-14-1	Bionordika (Germany)	CC-11765
vinblastine sulfate	143-67-9	Bionordika (Germany)	CC-11762

Supplementary Table 6. Standard mixture concentrations for LC-MS

	Concentrations of analytes (mg/L)		
	loganic acid, loganin, secologanin, strictosidine, tryptophan, tryptamine	tabersonine, vindoline, catharanthine, vinblastine	tetrahydroalstonine
LC-std-1	0.05	0.001	0.00025
LC-std-2	0.1	0.002	0.0005
LC-std-3	0.25	0.005	0.00125
LC-std-4	0.5	0.01	0.0025
LC-std-5	1	0.02	0.005
LC-std-6	2.5	0.05	0.0125
LC-std-7	5	0.1	0.025
LC-std-8	10	0.2	0.05
LC-std-9	25	0.5	0.125
LC-std-10	50	1	0.25

Supplementary Table 7. Metabolite retention times (RTs) and quantifier and qualifier fragments (EVOQ Elite triple quadrupole MS).

Metabolite	Molecular formula	Molecular weight	Precursor	Quantifier (CE [V])	Qualifer (CE [V])
caffeine (IS)	$C_8H_{10}N_4O_2$	194.08	195.1	138.0 [15]	110.0 [22]
loganic acid	$C_{16}H_{24}O_{10}$	376.14	377.1	214.6 [5]	178.8 [15]
loganin	$C_{17}H_{26}O_{10}$	390.15	391.2	229.0 [7]	179.0 [17], 211.0 [7]
secologanin	$C_{17}H_{24}O_{10}$	388.14	389.1	227.2 [5]	107.0 [10], 165.0 [7]
tryptophan	$C_{11}H_{12}N_2O_2$	204.09	205.1	146.0 [16]	188.0 [9], 118.1 [32]
tryptamine	$C_{10}H_{12}N_2$	160.10	161.1	144.1 [9]	117.1 [28], 115.0 [40]
strictosidine	$C_{27}H_{34}N_2O_9$	530.23	531.2	352.0 [25]	282.0 [30], 144.0 [35]
tetrahydroalstonine	$C_{21}H_{24}N_2O_3$	352.18	353.2	144.0 [25]	222.0 [18], 210.0 [18]
tabersonine	$C_{21}H_{24}N_2O_2$	336.18	337.2	305.1 [23]	228.0 [24], 136.1 [27]
catharanthine	$C_{21}H_{24}N_2O_2$	336.18	337.2	144.1 [19]	173.1 [14], 165.1 [19]
vindoline	$C_{25}H_{32}N_2O_6$	456.23	457.2	187.9 [30]	396.9 [20]
anhydrovinblastine*	$C_{46}H_{56}N_4O_8$	792.97	397.2	262.6 [27]	367.2 [18], 346.1 [24]
vinblastine	$C_{46}H_{58}N_4O_9$	810.42	406.2	271.6 [27]	355.1 [24], 376.1 [25]

* Authentic standard of anhydrovinblastine was unavailable. Exact mass and MSMS data were acquired using HRMS. The most abundant ions 262.6, 367.2 and 346.1 obtained by fragmentation of its double-charged ion (m/z 397.2) were comparable with the most abundant ions 271.6, 376.1 and 355.1 obtained by fragmentation of double-charged ion of its analogue vinblastine (m/z 406.2).

Supplementary Table 8. Standard mixture concentrations for GC-FID

	Concentrations of analytes (mg/L)	
	Citronellol, & geraniol (mg/L)	8-hydroxygeraniol (mg/L)
GC-std-1	10	40
GC-std-2	20	80
GC-std-3	40	160
GC-std-4	5	20
GC-std-5	2.5	10
GC-std-6	1.25	5
GC-std-7	0.5	2
GC-std-8	0.25	1

Supplementary Methods

Purification of catharanthine and vindoline

Fermentation broth samples were taken from each ambr®250 bioreactor, which were used to extract and purify catharanthine and vindoline by liquid-liquid extraction (LLE), thin-layer chromatography (TLC), and preparative-scale high performance liquid chromatography (HPLC).

To extract catharanthine and vindoline, 150 mL broth was first acidified to pH 2.0 by adding 99.9% formic acid and extracted three times with 100 mL hexane. The aqueous phase was separated and neutralized with 28-30% NH_4OH, then extracted three times with 150 mL dichloromethane in a 500 mL separatory funnel. The organic phase was combined and evaporated at 35 °C to dryness using a rotary evaporator. The dried organic extract from LLE was dissolved in methanol and separated by prep-TLC using (dichloromethane/ethyl acetate/triethylamine) (1/1/0.03) as development solvent. Vindoline and catharanthine bands were cut and soaked in methanol, sonicated then filtered, and the solvent was dried under reduced vacuum and then nitrogen flow. The resulting samples were dissolved in 400 µL methanol, which was further diluted with water to a final volume of 800 µL. The sample was subjected to a reverse-phase separation using a Thermo Scientific™ Dionex™ Ultimate™ 3000 HPLC coupled to a UV/Vis diode array detector (DAD) under the following conditions: column, Waters XBridge BEH C18 OBD Prep, 130 Å, 5 µm, 10 × 250 mm; column temperature, 30 °C, solvent A (water with 0.1% formic acid) and solvent B (methanol). The mobile phase was: isocratic 0-2 min at 10% B; gradient 2-20 min from 10 to 95% B; isocratic 20-25 min at 95% B. The column was re-equilibrated for 16 min prior to each injection. The flow rate was 3.0 mL/min. The fraction from 16.3 to 19.3 min where catharanthine and vindoline peaks eluted with UV λmax of 226, 214 and 254 nm was manually collected. The fraction was then dried by evaporating the solvent at room temperature for 10 h using a vacuum concentrator (SpeedVacTM SPD1030/2030, Thermo Fisher Scientific, Asheville, USA) and kept at -20 °C until the final chemical coupling reaction.

Analytical Methods

Liquid chromatography - mass spectrometry (LC-MS)

For the analysis of tetrahydroalstonine, strictosidine, secologanin, loganin and loganic acid, data were acquired on an Advance UHPLC system (Bruker Daltonics, Fremont, CA, USA) equipped with a binary pump, degasser and PAL HTC-xt autosampler (CTC Analytics AG, Switzerland) coupled to an EVOQ Elite

triple quadrupole mass spectrometer (Bruker Daltonics, Fremont, CA, USA). The system was operated with MS Workstation 8.2.1 software (Bruker Daltonics, Fremont, CA, USA). The analytical column was a 100 mm C18 Acquity UPLC HSS T3 column 100 Å, 1.8 µm particle size, 2.1 mm i.d. (Waters, Milford, MA, USA) with a KrudKatcher, HPLC in-line column filter, 0.5 µm x 0.004 in i.d. (Phenomenex, Torrance, CA, USA). The column oven temperature was set to 35 °C with an injection volume of 1 µL and a 2 µL injection loop. The mobile phase consisted of 0.1% formic acid in Milli-Q (solvent A) and 0.1% formic acid in acetonitrile (solvent B), delivered at a constant flow rate of 0.5 mL/min with the following gradient profile: the following gradient profile: isocratic 0-0.8 min at 5% B; gradient 0.8-4 min from 5 to 55% B; gradient 4-4.2 min from 55 to 90% B, isocratic 4.2-5.1 min at 90% B. The column was re-equilibrated for 2 min prior to each injection.

Acquisition was carried out using electrospray ionization operated in positive ion mode. The following parameters were used to acquire Multiple Reaction Monitoring (MRM) data: spray voltage: 4.5 kV, cone temperature: 350 °C, cone gas flow 20, probe gas flow: 50, nebulizer gas flow: 50, heated probe temperature: 300 °C, exhaust gas: on, CID: 1.5 mTorr. The MRM scan time was set to 30 ms with a standard resolution for all transitions. The collision energy (CE) was optimized for each transition. Analyte stock solutions were prepared in ethanol for geraniol, 8-hydroxygeraniol, cis-trans-nepetalactol, tryptamine, and Milli-Q water for 8-oxogeranial, loganic acid, loganin, secologanin, strictosidine, tetrahydroalstonine, tabersonine, catharanthine, vindoline and vinblastine sulfate. Aliquots of each solution were diluted with 0.1% formic acid for eight calibration levels (**Supplementary Table 6**).

High-resolution mass spectrometry

Detection of pathway intermediates downstream of strictosidine (strictosidine aglycone, etc) hydroxytabersonine/hydroxycatharanthine, 16-methoxytabersonine, 3-hydroxy-16-methoxy-2,3-dihydrotabersonine, desacetoxyvindoline, deacetylvindoline) were conducted by LC-MS measurements on a Dionex UltiMate 3000 UHPLC (Fisher Scientific, San Jose, CA) connected to an Orbitrap Fusion Mass Spectrometer (Thermo Fisher Scientific, San Jose, CA). The system used an Agilent Zorbax Eclipse Plus C18 2.1 x 100 mm, 1.8 µm column kept at 35 °C. The flow rate was 0.350 mL/min with 0.1% formic acid (A) and 0.1% formic acid in acetonitrile (B) as mobile phase. The gradient had 5% B until 1 min and then followed a linear gradient to 95% B within 5 min. This solvent composition was held for 1.5 min and then immediately changed to 5% B and held until 8 min. The sample (1 µL) was passed on to the MS equipped with a heated electrospray ionization source (HESI) in positive and negative-ion mode, spray voltage was 3500 V and 2500 V, respectively. Sheath gas was set to 50 (a.u.), aux gas to 10 (a.u.) and sweep gas to 1

(a.u.). The cone and probe temperatures were 325 °C and 380 °C, respectively. The resolution was set to 60000, scan range was 100 to 1000 Da and time between scans was 50 ms. Detection of masses that match with (< 10 ppm difference) the theoretical $[M+H]^+$ adduct for tabersonine (337.19105 Da), catharanthine (337.19105 Da), vindoline (457.23331 Da) and vinblastine (811.42766 Da and as $[M+2H]^{2+}$, 406.21780 Da) was conducted in full scan and the retention time confirmed with authentic standards (**Supplementary Table 7, Supplementary Fig. 9, 11-12**). Accurate mass match with < 10 ppm difference to expected mass of the conjugate acid of strictosidine aglycone (369.18089 Da), geissoschizine/16-hydroxytabersonine/hydroxycatharanthine (353.18597 Da), (iso)stemmadenine (355.20162 Da), stemmadenine acetate/ dihydroprecondylocarpine acetate (397.21218 Da), 16-methoxytabersonine (367.20162 Da), 3-hydroxy-16-methoxy-2,3-dihydrotabersonine (385.21218 Da), deacetoxyvidoline (399.22783 Da) and deacetylvinddoline (415.22275 Da) was detected but not confirmed with standards.

Gas Chromatography – Flame Ionization Detector (GC-FID)

For the analysis of geraniol, 8-hydroxygeraniol, data were acquired on a Thermo Scientific Trace 1300 gas chromatograph with a flame ionization detector (FID) and split/splitless injector and Chromeleon version 7.2.1 as operating software. A 30 m x 0.25 mm ID MXT-WAX Restek column with 0.25 µm film thickness (Bellefonte, PA, USA) was used. As a carrier gas Helium was used at a flow rate of 1 ml/min. The injector temperature was 250 °C, in split mode with a split flow of 10 mL/min, a split ratio of 1:10, and a purge flow of 5 mL/min. The injection volume was 1 µL and the initial oven temperature was 50 °C, held for 1.5 min and increased to 240 °C with 20 °C/min. The FID data was collected at a rate of 10 Hz with a detector temperature of 280 °C and the following gas settings: airflow 350 mL/min, makeup gas (helium) 40 mL/min, and hydrogen flow 35 mL/min. The air was cleaned with a UHP-35-ZA-S Parker Domnick Hunter (Gateshead, England) zero air generator and the hydrogen was created on-site with a 20HMD Parker Domnick Hunter (Gateshead, England) hydrogen generator. Aliquots of each analyte had eight calibration levels (**Supplementary Table 8**).

Supplementary Figures

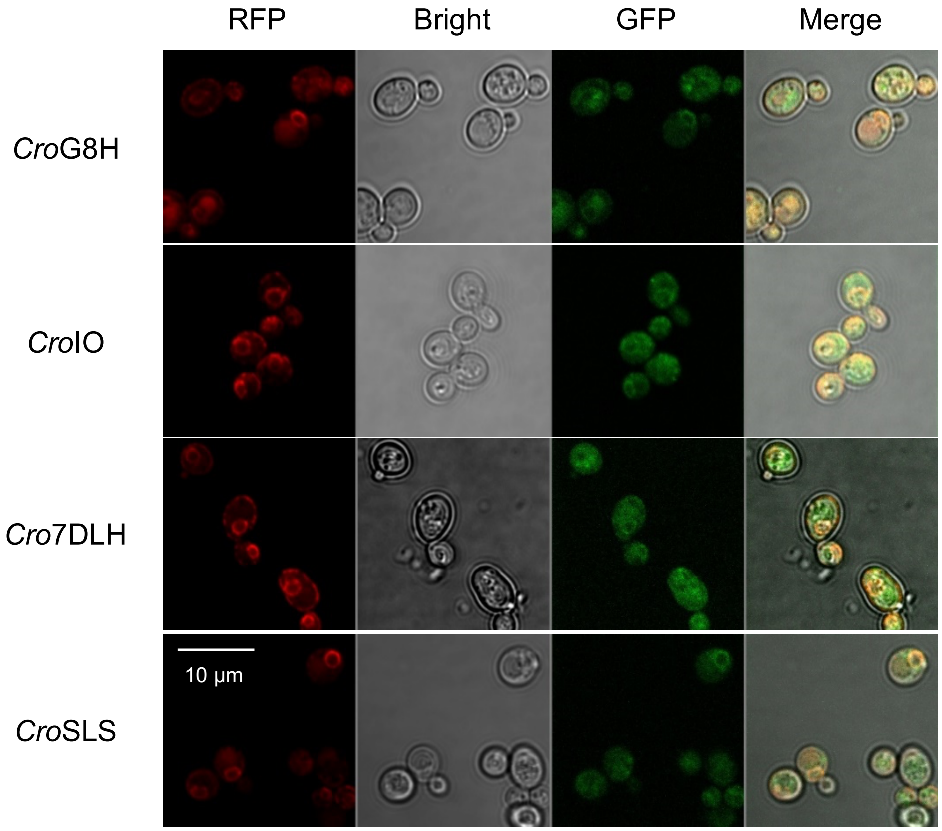

Supplementary Fig. 1│Subcellular localization of *Cro*G8H, *Cro*IO, *Cro*7DLH and *Cro*SLS. Each MIA gene was fused to a yEGFP (on the C-terminal) and expressed in the wild-type strain CEN.PK2-1C under the *TEF1* promoter. The red marker was SS-mCherry-HDEL which localizes to the ER. All yeast strains (*Cro*G8H, MIA-GF-17; *Cro*IO, MIA-GF-18; *Cro*7DLH, MIA-GF-19; *Cro*SLS, MIA-GF-20) were cultivated in the SC medium overnight (16-20 hours) and washed twice with the same medium before microscopy analysis. Images were acquired using a confocal microscope (magnification 63 x, white bar is 10 µm).

Single gene overexpression in parental strictosidine strain MIA-BG

The first strictosidine strain MIA-BG was able to produce 9.81 µg/L strictosidine, which is significantly less than the previously reported titer by engineered de novo strictosidine Strain 4[1]. To deduce potential bottleneck(s) in this pathway, we individually overexpressed all genes involved in the strictosidine pathway under the control of a constitutive promoter from a high copy (pESC-URA) plasmid (**Supplementary Table 4**). All resulting strains were cultivated in SC-Uracil medium supplemented with 1 mM tryptamine together with 0.1 mM each of the available precursors of strictosidine, namely geraniol, 8-hydroxygeraniol, 8-oxogeranial, *cis*-*trans*-nepetalactol, loganic acid, loganin, and secologanin. The cells were grown for 6 days and final broths were analyzed for the production of loganin, secologanin and strictosidine. No substantial improvement in strictosidine production was observed from single gene overexpression when fed intermediates in the upper part of the parthway, namely, geraniol, 8-hydroxygeraniol, 8-oxogeranial, cis-trans-nepetalactol, probably due to other bottlenecks in the lower part of the pathway that could potentially undermine their positive effect. Similarly, overexpression of all genes gave similar strictosidine production when fed with loganic acid or loganin, presumably due to poor permeability of these two precursors. Overexpression of t*Cro*STR resulted in a significantly improved strictosidine production and reduction in unused secologanin.

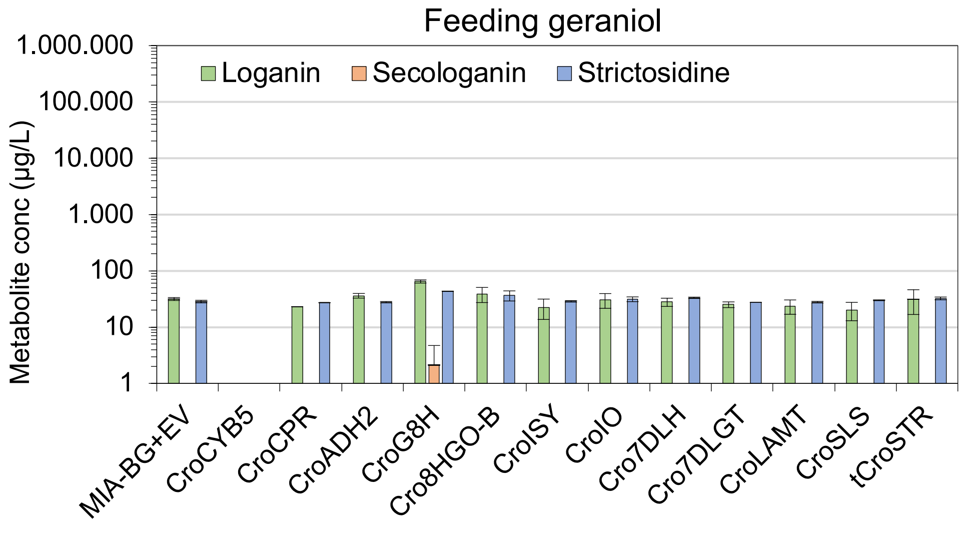

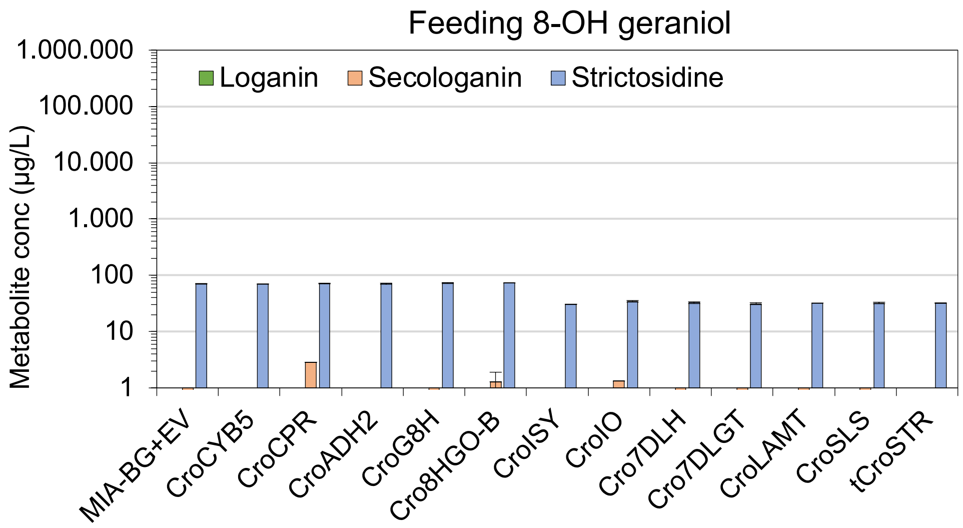
Feeding 8-OH geraniol
Loganin
Secologanin
Strictosidine
Metabolite conc (µg/L)
1.000.000
100.000
10.000
1.000
100
10
1
MIA-BG+EV
CroCYB5
CroCPR
CroADH2
CroG8H
Cro8HGO-B
CroISY
CroIO
Cro7DLH
Cro7DLGT
CroLAMT
CroSLS
tCroSTR

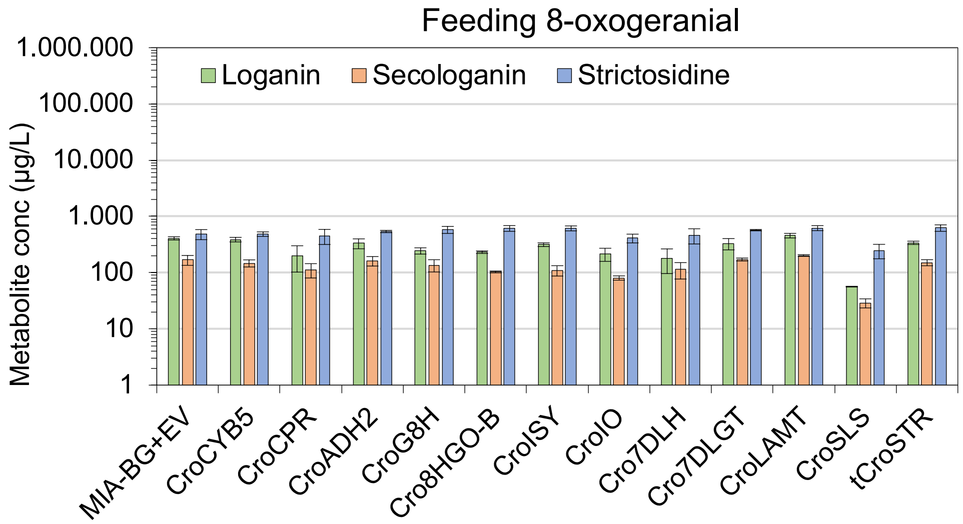
Feeding 8-oxogeranial
Loganin
Secologanin
Strictosidine
Metabolite conc (µg/L)
1.000.000
100.000
10.000
1.000
100
10
1
MIA-BG+EV
CroCYB5
CroCPR
CroADH2
CroG8H
Cro8HGO-B
CroISY
CroIO
Cro7DLH
Cro7DLGT
CroLAMT
CroSLS
tCroSTR

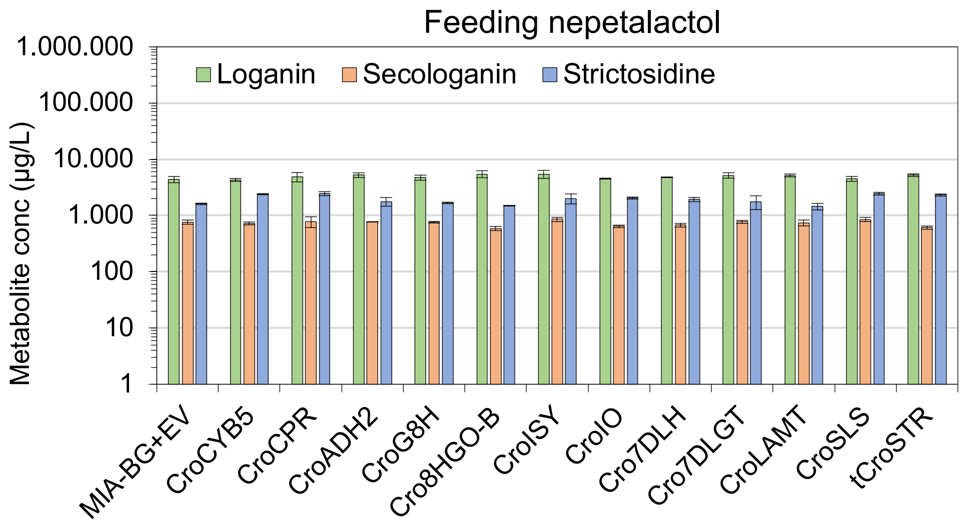
Feeding nepetalactol
Loganin
Secologanin
Strictosidine
Metabolite conc (µg/L)
1.000.000
100.000
10.000
1.000
100
10
1
MIA-BG+EV
CroCYB5
CroCPR
CroADH2
CroG8H
Cro8HGO-B
CroISY
CroIO
Cro7DLH
Cro7DLGT
CroLAMT
CroSLS
tCroSTR

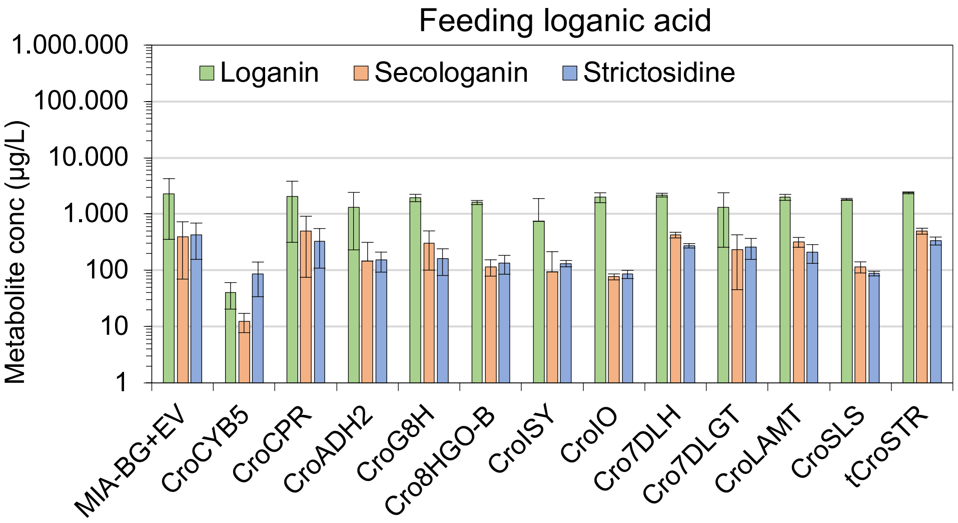
Feeding loganic acid
Loganin
Secologanin
Strictosidine
Metabolite conc (µg/L)
1.000.000
100.000
10.000
1.000
100
10
1
MIA-BG+EV
CroCYB5
CroCPR
CroADH2
CroG8H
Cro8HGO-B
CroISY
CroIO
Cro7DLH
Cro7DLGT
CroLAMT
CroSLS
tCroSTR

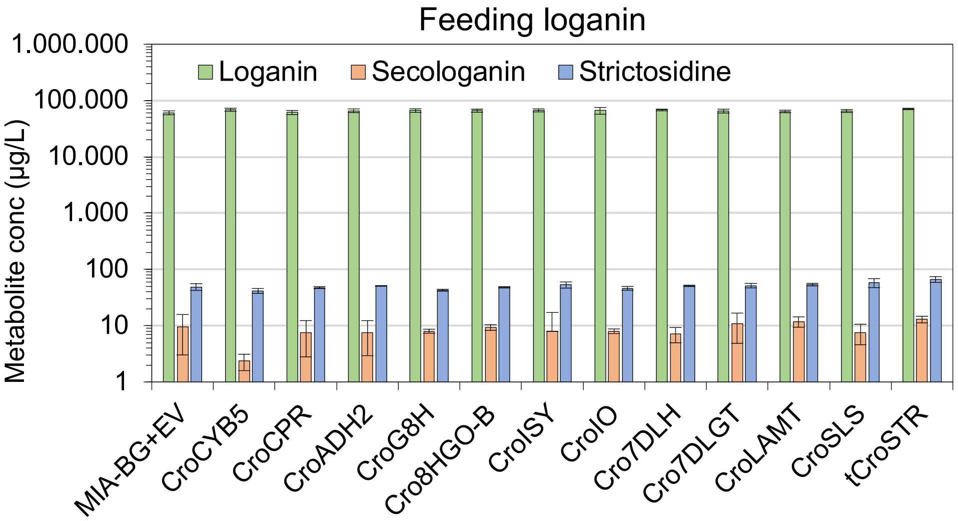
Feeding loganin
Loganin
Secologanin
Strictosidine
Metabolite conc (µg/L)
1.000.000
100.000
10.000
1.000
100
10
1
MIA-BG+EV
CroCYB5
CroCPR
CroADH2
CroG8H
Cro8HGO-B
CroISY
CroIO
Cro7DLH
Cro7DLGT
CroLAMT
CroSLS
tCroSTR

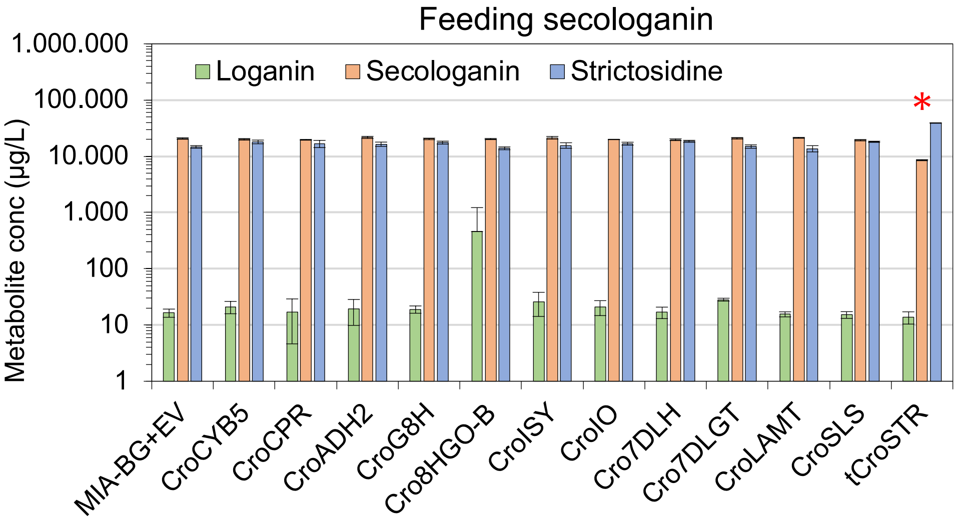
Feeding secologanin
Loganin
Secologanin
Strictosidine
*
Metabolite conc (µg/L)
1.000.000
100.000
10.000
1.000
100
10
1
MIA-BG+EV
CroCYB5
CroCPR
CroADH2
CroG8H
Cro8HGO-B
CroISY
CroIO
Cro7DLH
Cro7DLGT
CroLAMT
CroSLS
tCroSTR

Supplementary Fig. 2 | Production of strictosidine in the MIA-BG strain with single-gene overexpression using high copy plasmid. Data presented are mean ± s.d. (n = 3). * p-value < 0.01. Student's two-tailed t-test.

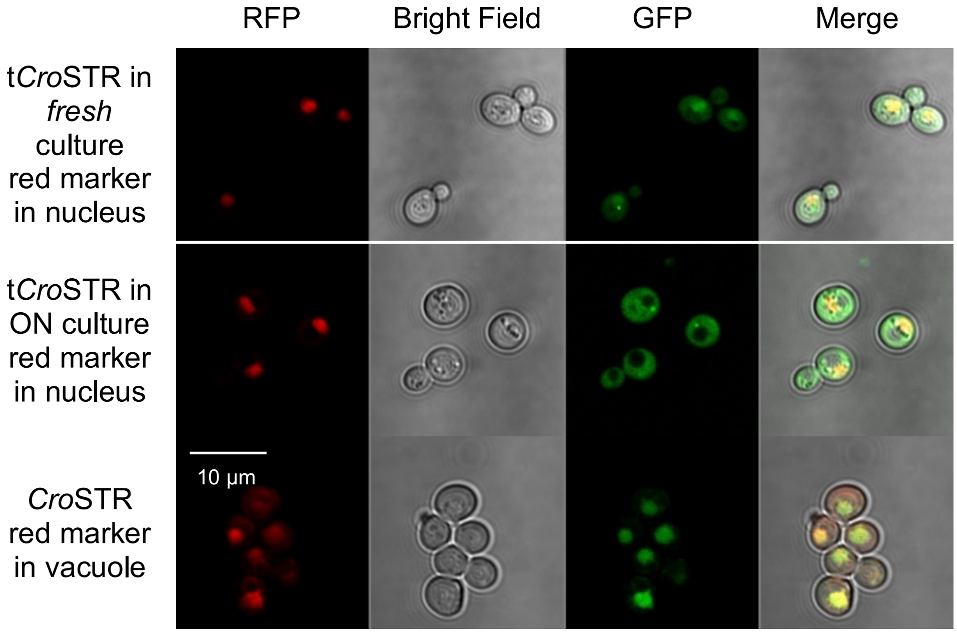

Supplementary Fig. 3 | Subcellular localization of truncated and full *Cro*STR. Each MIA gene was fused to a yEGFP (on the C-terminal) and expressed in the wild-type strain CEN.PK2-1C under the P_{TEF1} promoter. For t*Cro*STR (MIA-GF-21-A), the red marker was NAB2-mCherry which localizes to the nucleus. t*Cro*STR in the fresh culture was localized in the nucleus, and in the overnight culture shifted to the cytoplasm. For *Cro*STR (MIA-GF-21-B), the red marker was VPH1-mCherry which localizes in the vacuole. All yeast strains were cultivated in the SC medium overnight (16-20 hours) and washed twice with the same medium before microscopy analysis. For t*Cro*STR, a fresh culture (4 hours) was also used for the microscopy analysis. Images were acquired using a confocal microscope (magnification 63 x, white bar is 10 μm).

Benchmark with previously reported *de novo* strictosidine strains

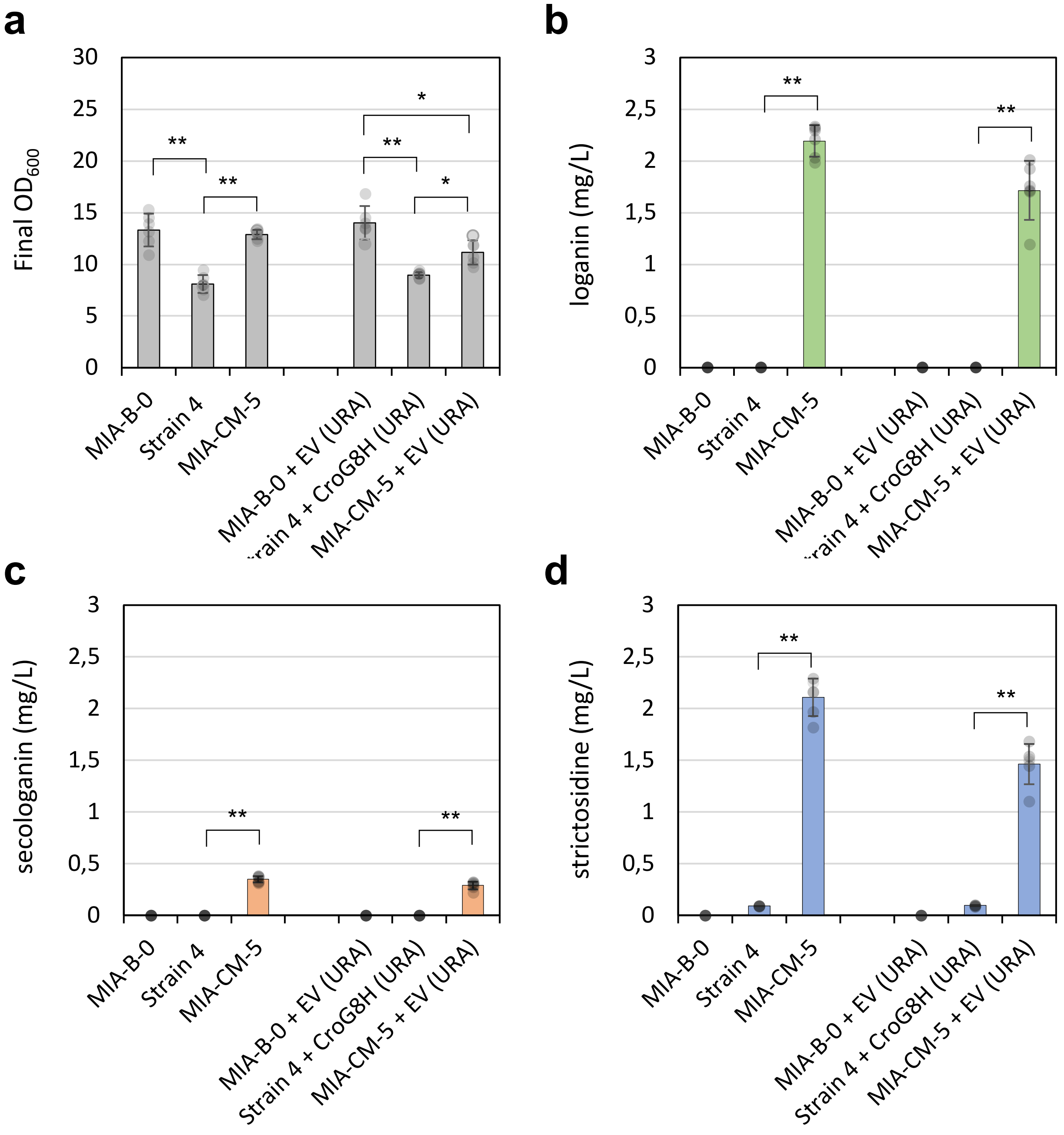

Supplementary Fig. 4 | Benchmark of the optimized *de novo* strictosidine strain MIA-CM-5 to previously reported strain. Strain 4 is the *de novo* strictosidine strain reported by Brown et al.[1]. Data presented are mean ± s.d. (n = 3). * *p*-value < 0.01, ** *p*-value < 0.0001.

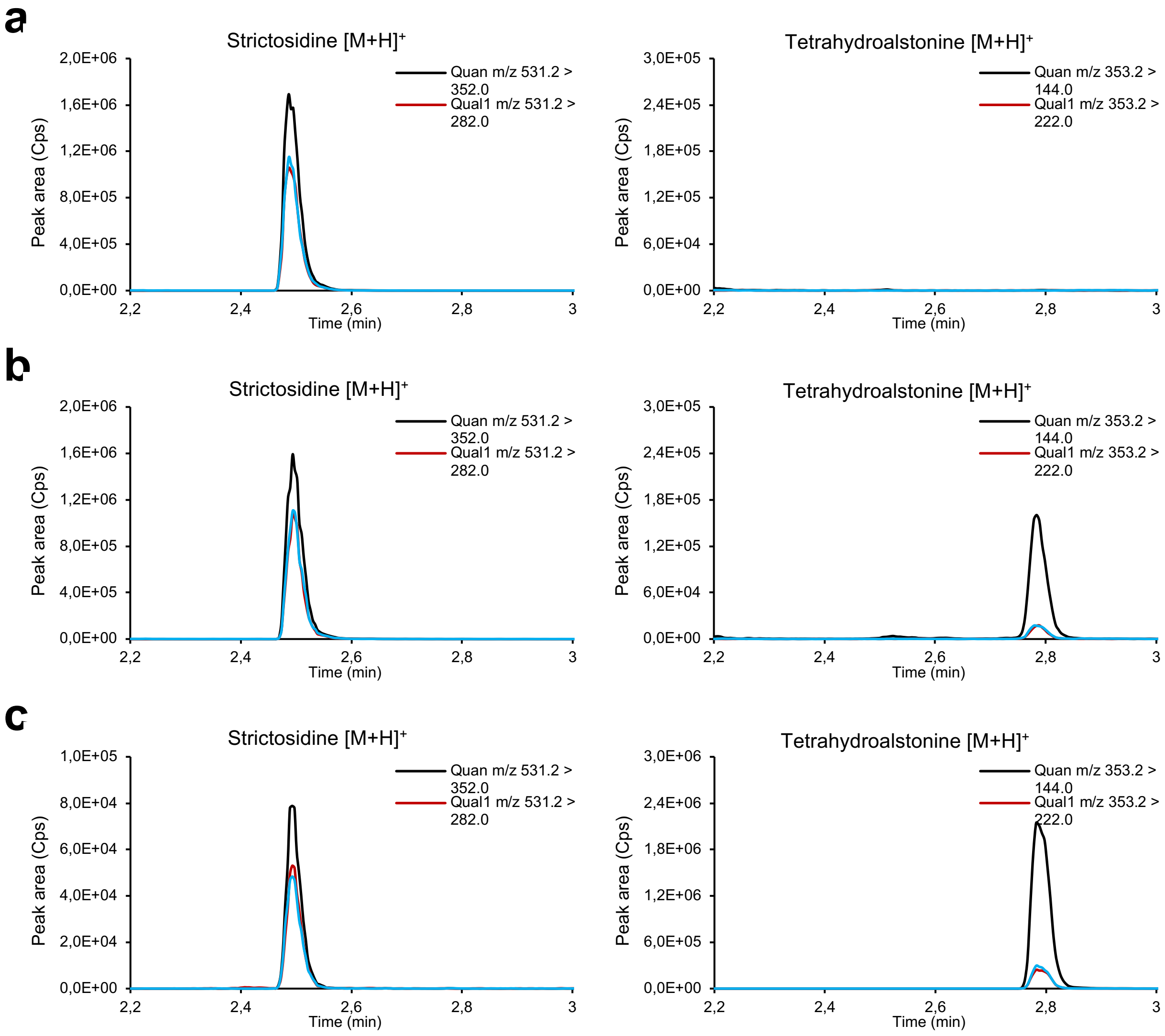

Supplementary Fig. 5 | Chromatogram of tetrahydroalstonine. a. strain MIA-CA-1 expressing *Cro*SGD and *Cro*THAS; **b.** strain MIA-CA-2, expressing *Rse*SGD and *Cro*THAS; **c.** authentic standards. Both MIA-CA-1 and MIA-CA-2 strains were cultivated in SC medium supplemented with 0.05 mM secologanin and 1 mM tryptamine for 6 days.

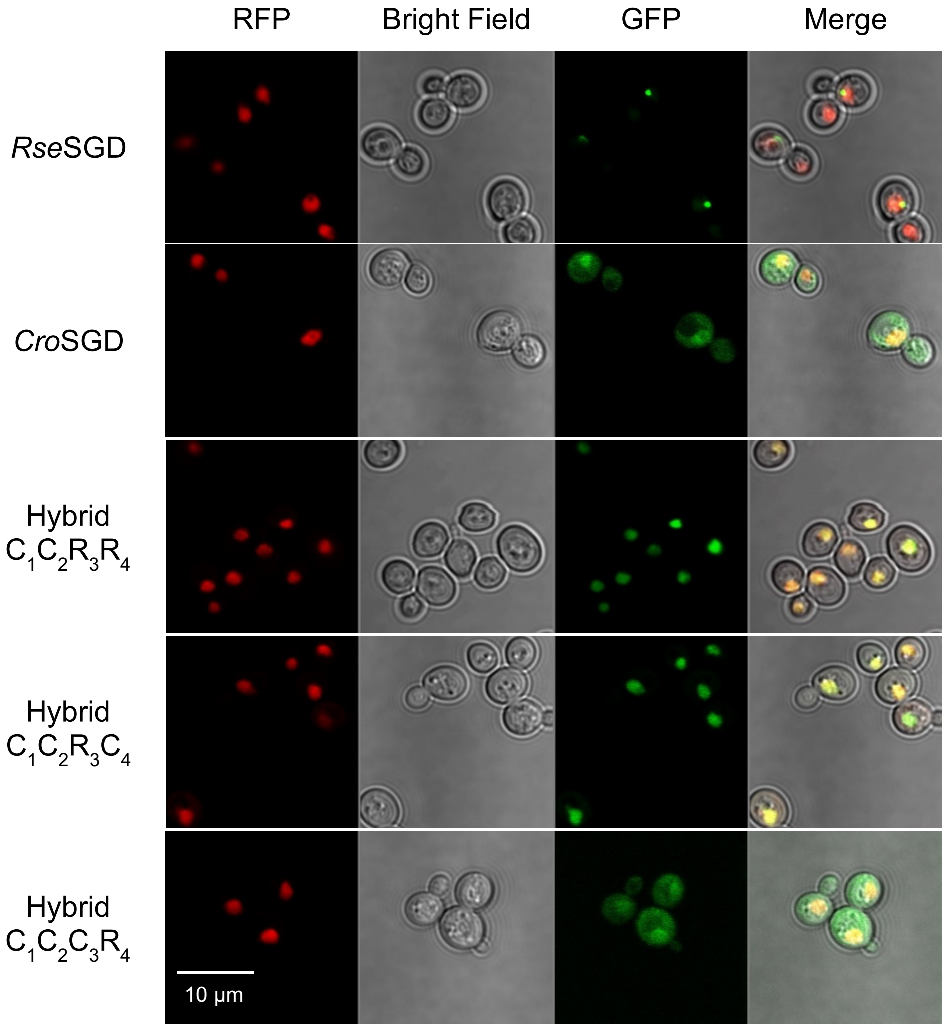

Supplementary Fig. 6│Subcellular localization of *Rse*SGD, *Cro*SGD, and hybrid variants. Each SGD gene or variant was fused to a yEGFP (on the N-terminal) and expressed in the wild-type strain CEN.PK2-1C under the P_{TEF1} promoter. For all strains the red marker was NAB2-mCherry which localizes to the nucleus. All yeast strains (*Rse*SGD, MIA-GF-22-A; *Cro*SGD, MIA-GF-22-B; Hybrid SGD $C_1C_2R_3R_4$, MIA-GF-22-C; Hybrid SGD $C_1C_2R_3C_4$, MIA-GF-22-D; Hybrid SGD $C_1C_2C_3R_4$, MIA-GF-22-E) were cultivated in the SC medium overnight (16-20 hours) and washed twice with the same medium before microscopy analysis. Images were acquired using a confocal microscope (magnification 63 x, Bar, 10 µm).

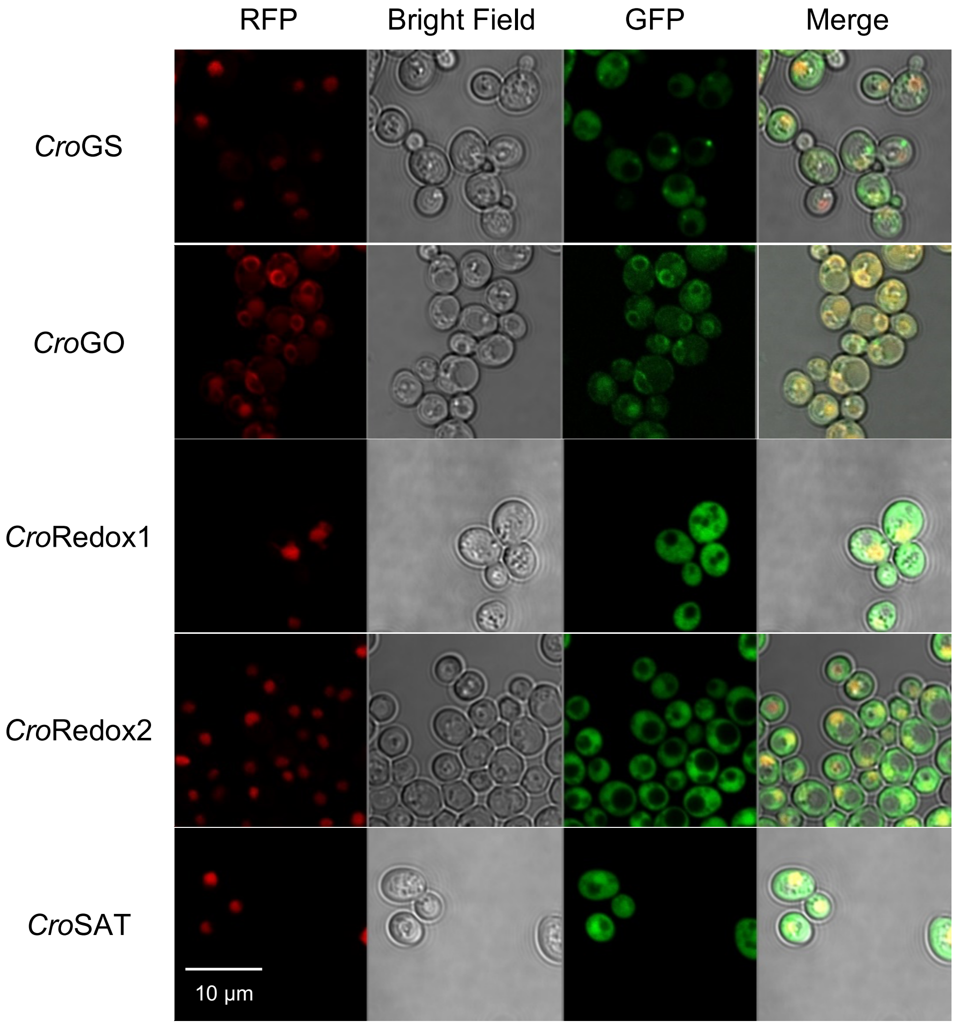

RFP
Bright Field
GFP
Merge
*Cro*GS
*Cro*GO
*Cro*Redox1
*Cro*Redox2
*Cro*SAT
10 μm

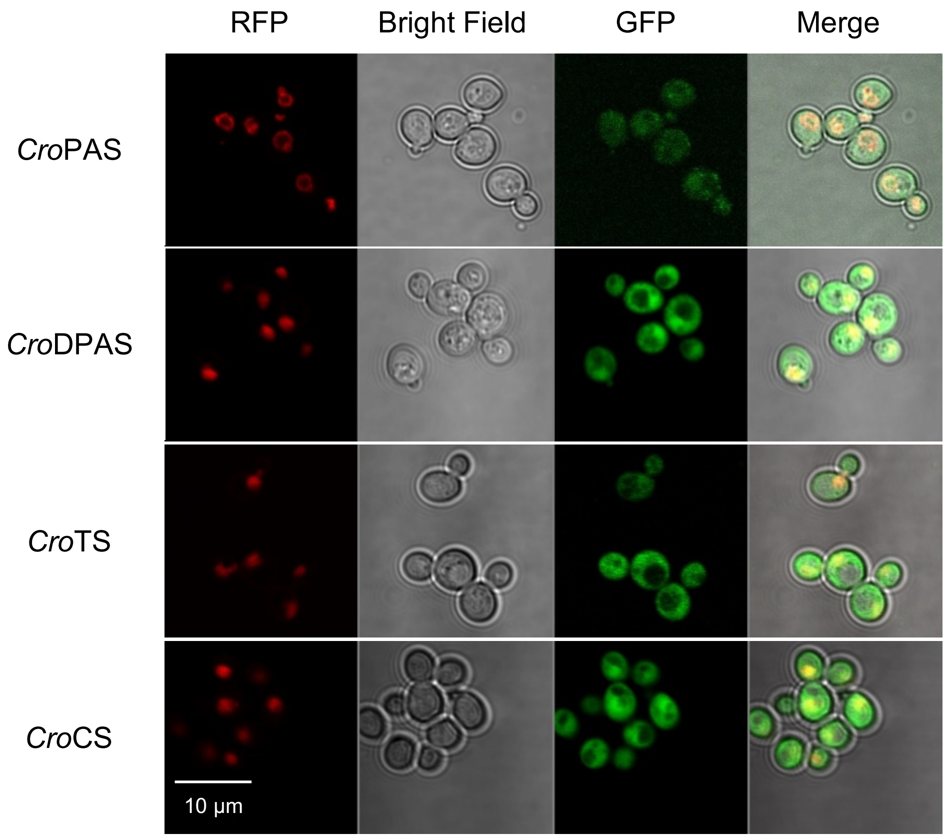

Supplementary Fig. 7│Subcellular localization of *Cro*GS, *Cro*GO, *Cro*Redox1, *Cro*Redox2, *Cro*SAT, *Cro*PAS, *Cro*DPAS, *Cro*TS and *Cro*CS. Each MIA gene was fused to a yEGFP (on the C-terminal) and expressed in the wild-type strain CEN.PK2-1C under the P_{TEF1} promoter. For all strains, the red marker was NAB2-mCherry which localizes to the nucleus, except for *Cro*GO and *Cro*PAS strains, in which the red markers were SS-mCherry-HDEL and VPH1-mCherry, which localize on the ER membrane and vacuole, respectively. All yeast strains (*Cro*GS, MIA-GF-01; *Cro*GO, MIA-GF-02; *Cro*Redox1, MIA-GF-03; *Cro*Redox2, MIA-GF-04; *Cro*SAT, MIA-GF-05; *Cro*PAS, MIA-GF-06; *Cro*DPAS, MIA-GF-07; *Cro*TS, MIA-GF-08; *Cro*CS, MIA-GF-09) were cultivated in the SC medium overnight (16-20 hours) and washed twice with the same medium before microscopy analysis. Images were acquired using a confocal microscope (magnification 63 x, white bar is 10 μm).

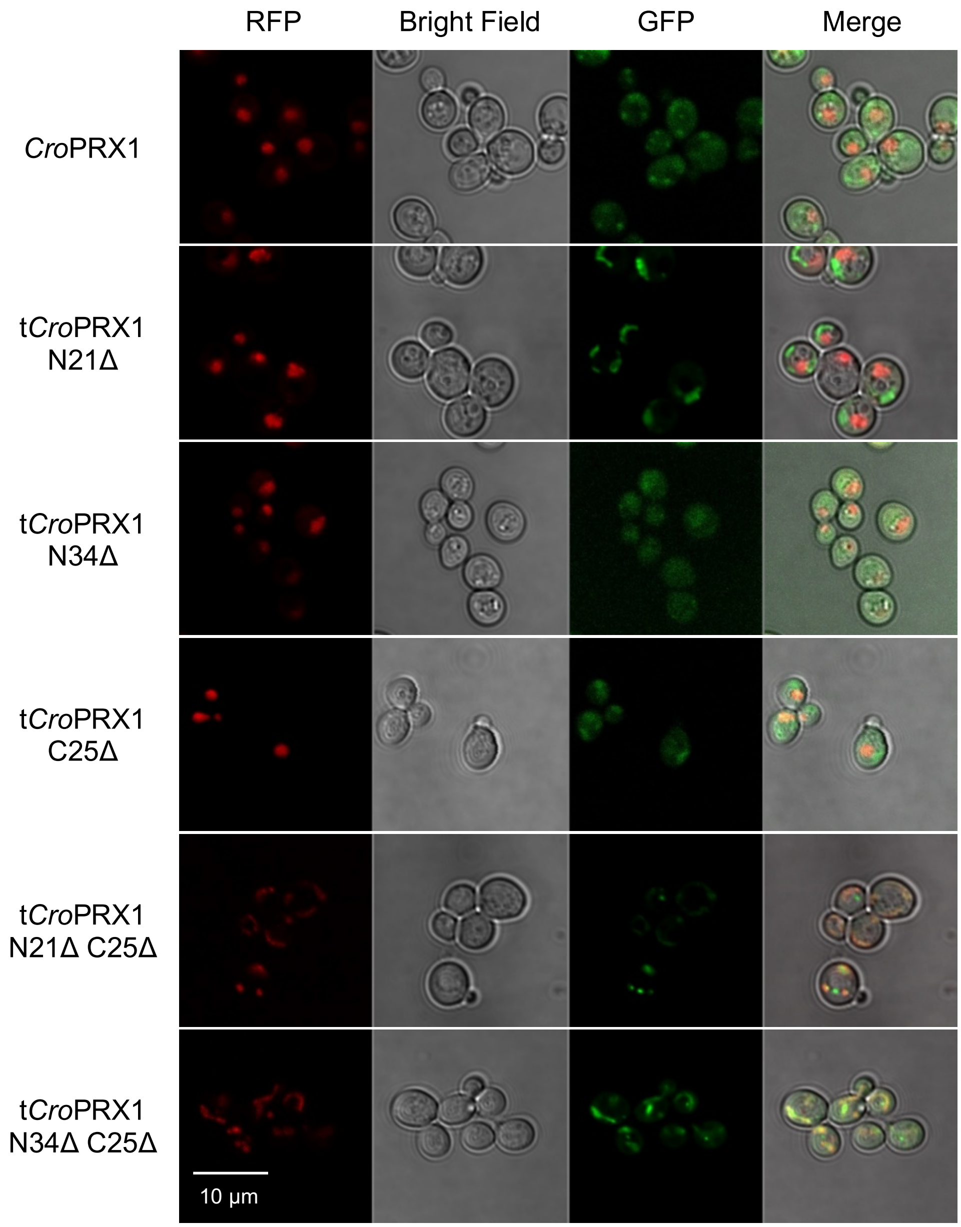

Supplementary Fig. 8│Subcellular localization of CroPRX1 and truncated variants. Each MIA gene was fused to a yEGFP (on the C-terminal) and expressed in the wild-type strain CEN.PK2-1C under the P_{TEF1} promoter. For *Cro*PRX1 (MIA-GF-10-A), t*Cro*PRX1$_{N34\Delta}$ (MIA-GF-10-C) and t*Cro*PRX1$_{C25\Delta}$ (MIA-GF-10-D), the red marker was NAB2-mCherry which localizes in the nucleus. For t*Cro*PRX1$_{N21\Delta\ (MIA\text{-}GF\text{-}10\text{-}B)}$ t*Cro*PRX1$_{N21\Delta C25\Delta}$ (MIA-GF-10-E) and t*Cro*PRX1$_{N34\Delta C25\Delta}$ (MIA-GF-10-F), the red marker was COX4-DuDre which localizes in the mitochondria. All yeast strains were cultivated in the SC medium overnight (16-20 hours) and washed twice with the same medium before microscopy analysis. Images were acquired using a confocal microscope (magnification 63 x, white bar is 10 μm).

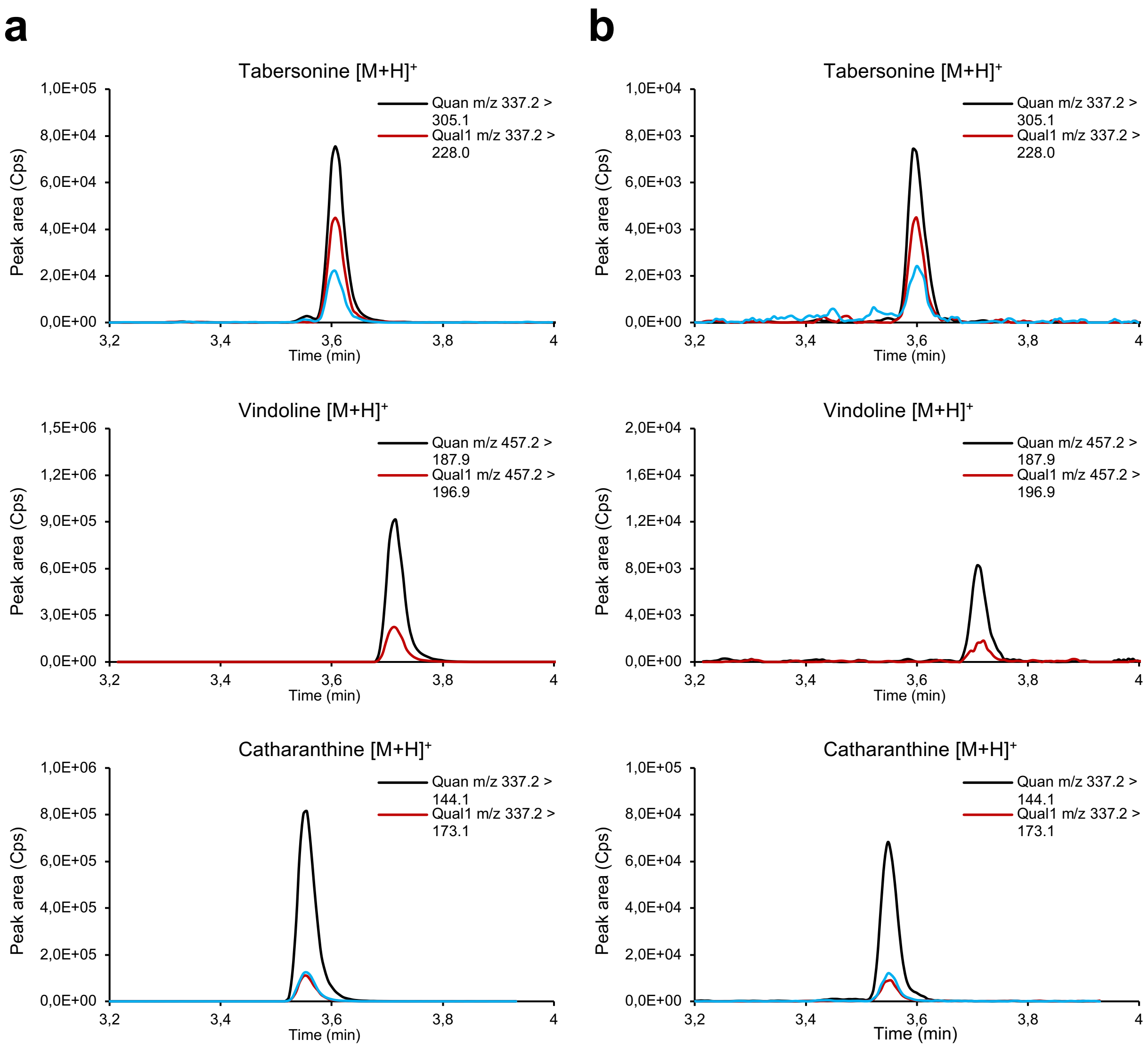

Supplementary Fig. 9│Chromatogram of tabersonine, catharanthine, and vindoline. a. Authentic standards; **b.** strain MIA-EM-2, cultivated in 3xSC (2% glucose) + 3 mM tryptophan for 7 days (spiked with 1% galactose on day 5).

Physiological characterization of engineered strains

The maximum growth rates for the wild type (CEN.PK2-1C) and all engineered yeast strains were characterized on the Growth Profiler 960 (EnzyScreen, Netherlands). All strains were grown in standard synthetic complete (SC) medium with 2% glucose as the carbon source for 3 days. The raw image data were processed using the manufacturer's software (GP960 Viewer) and converted into G-values, which were further converted into OD_{600} values equivalent to those obtained using a standard curve measured on a spectrophotometer. The maximum growth rate for each colony was determined by linear regression of the OD_{600} values during the mid-exponential growth phase ($R^2 > 0.95$).

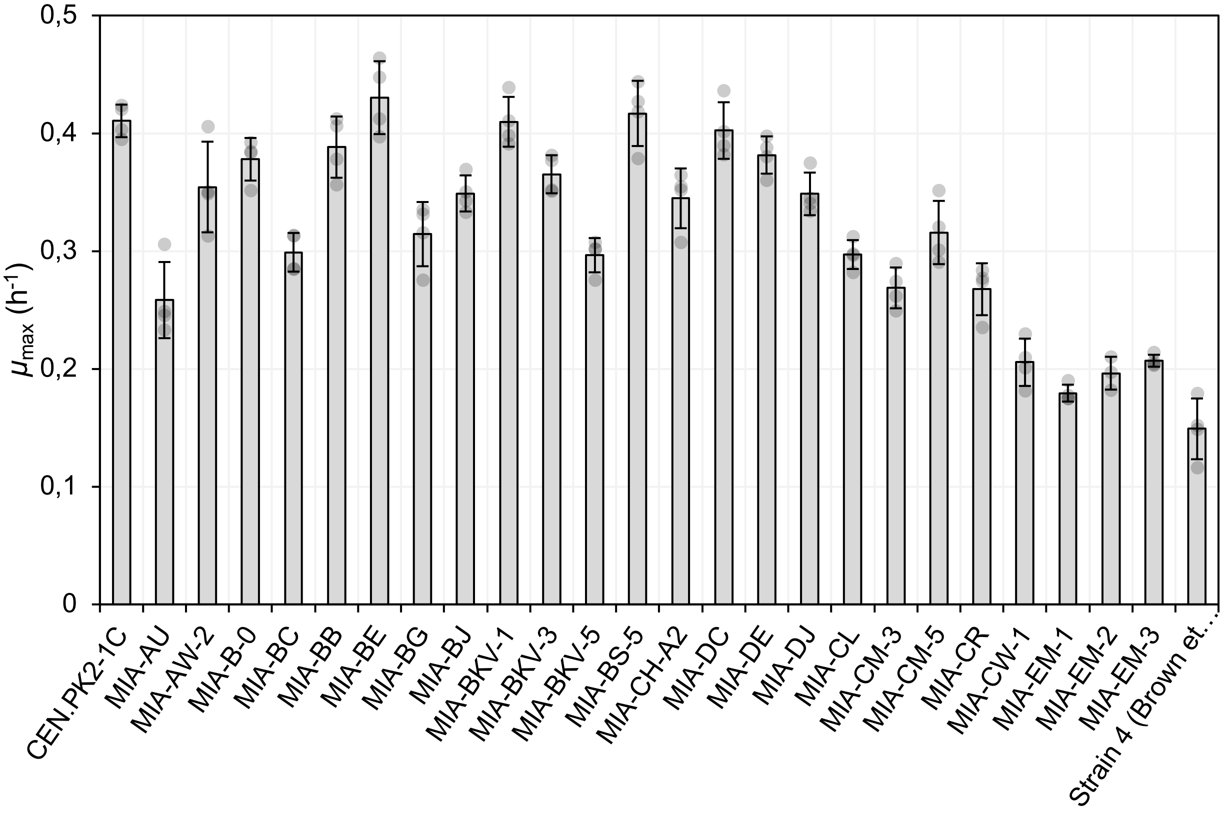

Supplementary Fig. 10 | Maximum specific growth rate (μ_{max}) of wild type and engineered MIA strains. All yeast strains were cultivated in Growth Profiler GP960 and data were analyzed using the manufacturer's software. Genotype information of all strains is listed in **Supplementary Table 2**. Data presented are mean ± s.d. (n = 4).

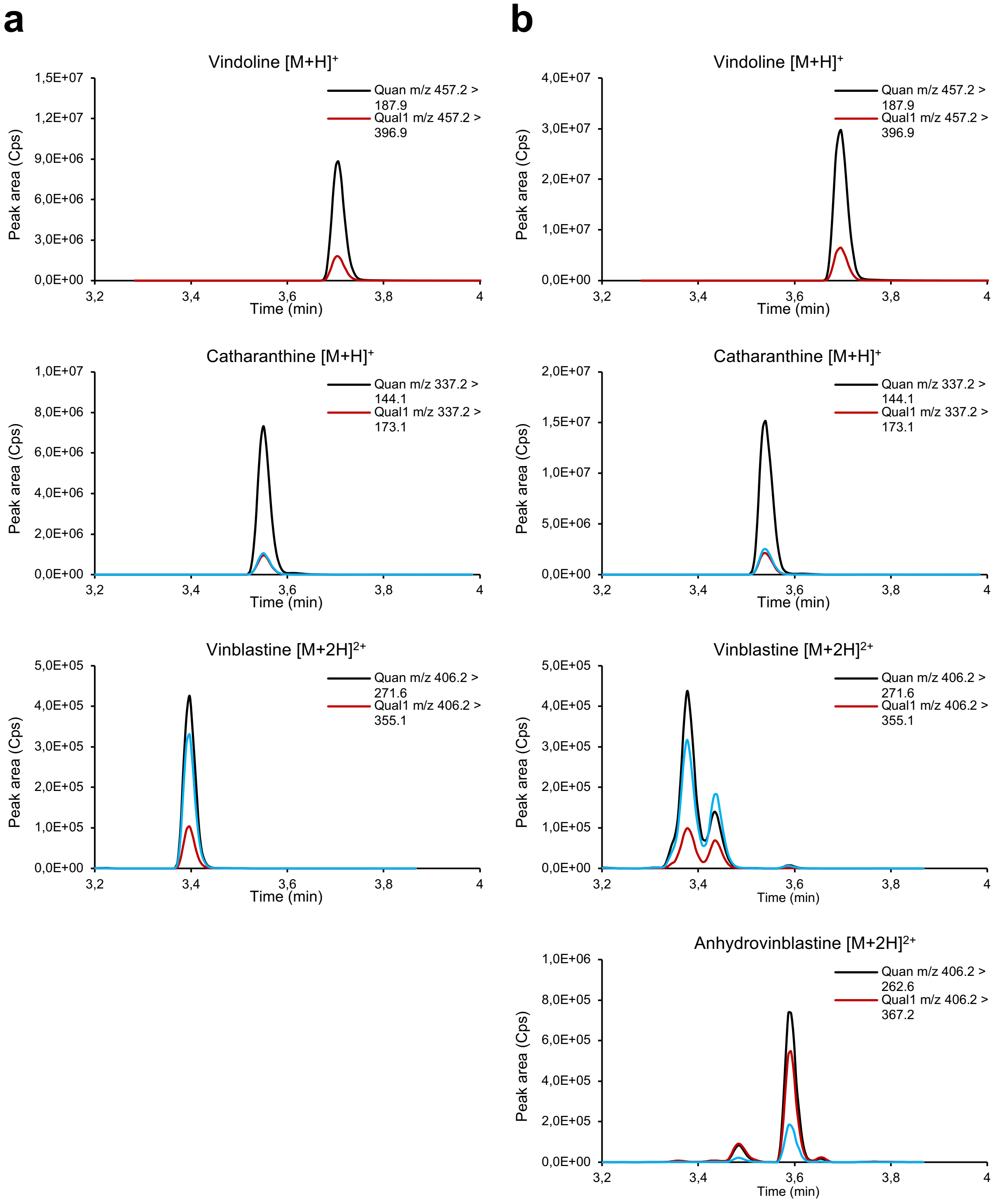

Supplementary Fig. 11│ Chromatograms of vinblastine and anhydrovinblastine. a. Authentic standard of vindoline, catharanthine and vinblastine**; b.** Vinblastine and anhydrovinblastine synthesized in the photo-chemical coupling reaction using pure catharanthine and vindoline standards.

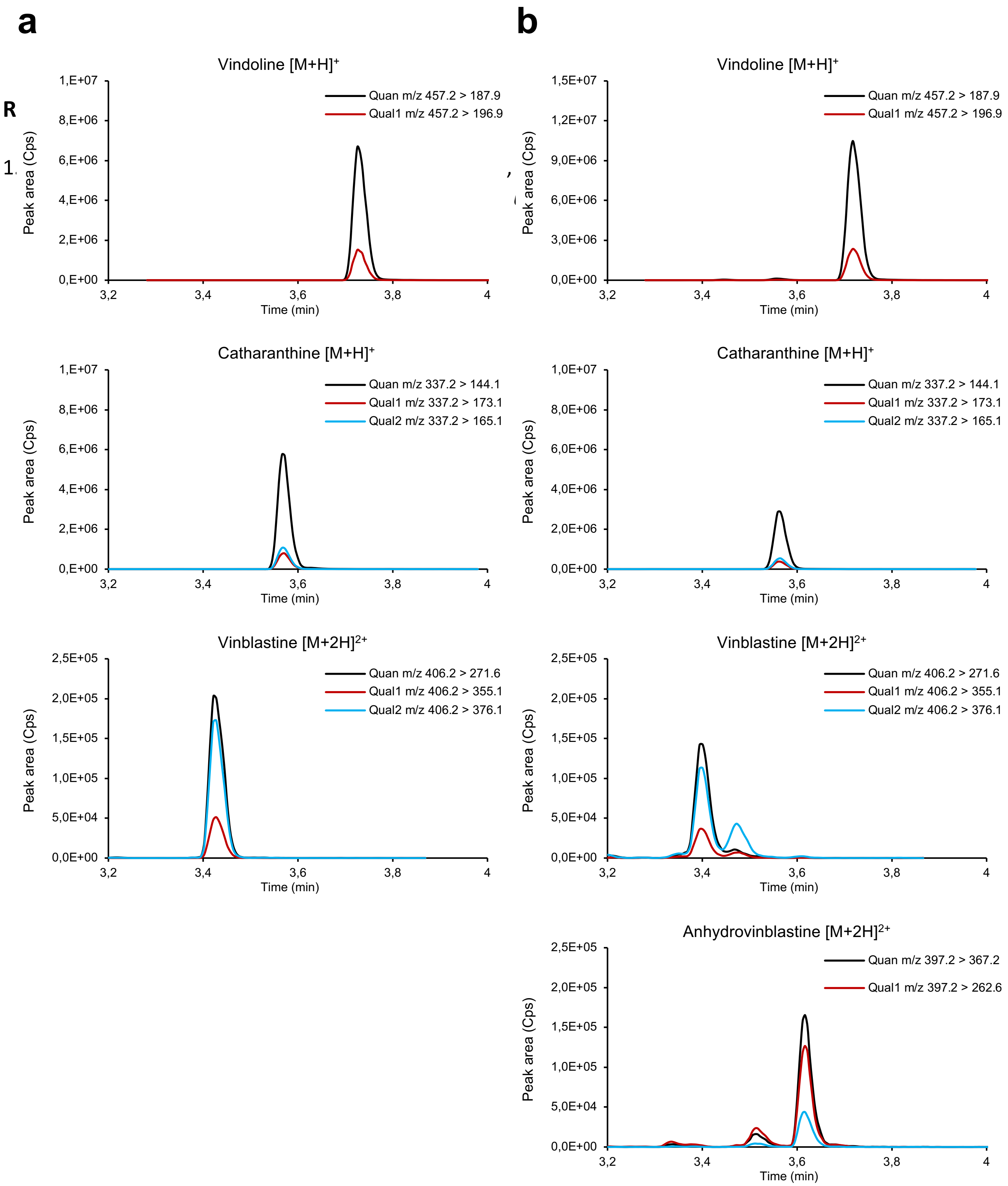

Supplementary Fig. 12 | Chromatograms of vinblastine and anhydrovinblastine. a. Authentic standard of vinblastine; **b.** Vinblastine and anhydrovinblastine synthesized by Fe^{3+}-catalyzed chemical coupling using pure catharanthine and vindoline standards. Authentic standard of anhydrovinblastine was

unavailable, yet the most abundant ions 262.6, 367.2 and 346.1 obtained by fragmentation of its double-charged ion (m/z 397.2) were comparable with the most abundant ions 271.6, 376.1 and 355.1 obtained by fragmentation of double-charged ion of its analogue vinblastine (m/z 406.2).

References

1. Burke, C. & Croteau, R. Geranyl diphosphate synthase from Abies grandis: cDNA isolation, functional expression, and characterization. *Arch. Biochem. Biophys.* **405**, 130–136 (2002).
2. Stanley Fernandez, S. M., Kellogg, B. A. & Poulter, C. D. Farnesyl diphosphate synthase. Altering the catalytic site to select for geranyl diphosphate activity. *Biochemistry* **39**, 15316–15321 (2000).
3. Ignea, C., Pontini, M., Maffei, M. E., Makris, A. M. & Kampranis, S. C. Engineering monoterpene production in yeast using a synthetic dominant negative geranyl diphosphate synthase. *ACS Synth. Biol.* **3**, 298–306 (2014).
4. Zhao, J. *et al.* Dynamic control of ERG20 expression combined with minimized endogenous downstream metabolism contributes to the improvement of geraniol production in Saccharomyces cerevisiae. *Microbial Cell Factories* vol. 16 (2017).
5. Jensen, N. B. *et al.* EasyClone: method for iterative chromosomal integration of multiple genes Saccharomyces cerevisiae. *FEMS Yeast Res.* **14**, 238–248 (2014).
6. Zhu, J. *et al.* A Validated Set of Fluorescent-Protein-Based Markers for Major Organelles in Yeast (Saccharomyces cerevisiae). *mBio* vol. 10 (2019).
