# Supplementary material for: A microbial supply chain for production of the anti-cancer drug vinblastine
Source: Nature. 2022 Aug 31;609(7926):341–7. doi: 10.1038/s41586-022-05157-3 (PMC9452304; doi:10.1038/s41586-022-05157-3)
Supplement: Supplementary file 2 — Reporting Summary [file 41586_2022_5157_MOESM2_ESM.pdf]

nature portfolio

Corresponding author(s): Michael K. Jensen and Jay D. Keasling

Last updated by author(s): Jun 6, 2022

Reporting Summary

Nature Portfolio wishes to improve the reproducibility of the work that we publish. This form provides structure for consistency and transparency in reporting. For further information on Nature Portfolio policies, see our Editorial Policies and the Editorial Policy Checklist.

Statistics

For all statistical analyses, confirm that the following items are present in the figure legend, table legend, main text, or Methods section.

n/a	Confirmed	
☐	☒	The exact sample size (*n*) for each experimental group/condition, given as a discrete number and unit of measurement
☐	☒	A statement on whether measurements were taken from distinct samples or whether the same sample was measured repeatedly
☐	☒	The statistical test(s) used AND whether they are one- or two-sided<br>*Only common tests should be described solely by name; describe more complex techniques in the Methods section.*
☒	☐	A description of all covariates tested
☒	☐	A description of any assumptions or corrections, such as tests of normality and adjustment for multiple comparisons
☐	☒	A full description of the statistical parameters including central tendency (e.g. means) or other basic estimates (e.g. regression coefficient) AND variation (e.g. standard deviation) or associated estimates of uncertainty (e.g. confidence intervals)
☒	☐	For null hypothesis testing, the test statistic (e.g. *F*, *t*, *r*) with confidence intervals, effect sizes, degrees of freedom and *P* value noted<br>*Give P values as exact values whenever suitable.*
☒	☐	For Bayesian analysis, information on the choice of priors and Markov chain Monte Carlo settings
☒	☐	For hierarchical and complex designs, identification of the appropriate level for tests and full reporting of outcomes
☒	☐	Estimates of effect sizes (e.g. Cohen's *d*, Pearson's *r*), indicating how they were calculated

Our web collection on statistics for biologists contains articles on many of the points above.

Software and code

Policy information about availability of computer code

Data collection	The targeted proteomics data were acquired with Agilent MassHunter Workstation Software version B.08.2, LC/MS Data Acquisition operating in dynamic MRM mode. MRM transitions for the targeted proteins were generated by Skyline software 20.2 (MacCoss Lab Software) and selection criteria excluded peptides with Met/Cys residues, tryptic peptides followed by additional cut sites (KK/RR), and peptides with praline adjacent to K/R cut sites. The data and Skyline methods are available on Panoramaweb (https://panoramaweb.org/microbial-synthesis-of-vinblastine. url).Proteins from the MIA-DJ (tabersonine-vinblastine double module) strain were analyzed using Cap-LC system equipped with a C18 easy spray column (Thermo Fisher Scientific, MA), coupled to Orbitrap Q Exactive HF-X mass spectrometer (Thermo Fisher Scientific) (Wright et al. 2020).
Data analysis	The untargeted proteomics data from MIA-DJ strain were analyzed using Proteome Discover 2.3 (Thermo Fisher Scientific) by searching against the S. cerevisiae proteome data (Uniprot ID UP000002311) combined with all heterologous protein sequences. The abundance of each protein is reported as the relative intensity with respect to total intensity of all identified peptides. For GC-FID data analysis Chromeleon version 7.2.1 was used, while LC-MS data was analysed using the MS Workstation 8.2.1 software.

For manuscripts utilizing custom algorithms or software that are central to the research but not yet described in published literature, software must be made available to editors and reviewers. We strongly encourage code deposition in a community repository (e.g. GitHub). See the Nature Portfolio guidelines for submitting code & software for further information.

Data

Policy information about availability of data

All manuscripts must include a data availability statement. This statement should provide the following information, where applicable:

- Accession codes, unique identifiers, or web links for publicly available datasets
- A description of any restrictions on data availability
- For clinical datasets or third party data, please ensure that the statement adheres to our policy

All metabolite data shown in figures and extended data figures are available as Source Data. Accession of all heterologous genes used in this study are listed in Supplementary Table 1. Targeted proteomics data for strain MIA-AU are available at ProteomeXchange with identifier PXD024976 (https://panoramaweb.org/Panorama%20Public/2021/JBEI%20-%20J.%20Zhang%20et%20al.%20-%20vinblastine%20paper/project-begin.view?). Untargeted proteomics data for strain MIA-DJ are available at ProteomeXchange with identifier PXD025067.

Human research participants

Policy information about studies involving human research participants and Sex and Gender in Research.

Reporting on sex and gender	N/A
Population characteristics	N/A
Recruitment	N/A
Ethics oversight	N/A

Note that full information on the approval of the study protocol must also be provided in the manuscript.

Field-specific reporting

Please select the one below that is the best fit for your research. If you are not sure, read the appropriate sections before making your selection.

☒ Life sciences ☐ Behavioural & social sciences ☐ Ecological, evolutionary & environmental sciences

For a reference copy of the document with all sections, see nature.com/documents/nr-reporting-summary-flat.pdf

Life sciences study design

All studies must disclose on these points even when the disclosure is negative.

Sample size	All metabolites and proteins presented in Figures and Extended Data Figures were from 3-6 biological replicates (i.e., independent culture from individual colonies). Based on standard deviation among replicates we regard this number of biological replicates to represent a valid trade-off between approximating the true mean and instrumentation band-width.
Data exclusions	No data were excluded from analysis. All data used can be found in Source data.
Replication	All measurements of metabolites and proteins were performed with 3-6 biological replicates (i.e., independent culture from individual colonies). All attempts of replication were included in the manuscript, and as such successfully used to draw conclusions based on statistical testing.
Randomization	Randomization was applied by randomly selecting colonies.
Blinding	Beyond randomization of colony picking, no blinding was applied as the data generated from random colony picking is expected to approach or even represent population means.

Reporting for specific materials, systems and methods

We require information from authors about some types of materials, experimental systems and methods used in many studies. Here, indicate whether each material, system or method listed is relevant to your study. If you are not sure if a list item applies to your research, read the appropriate section before selecting a response.

Materials & experimental systems

n/a	Involved in the study
☒	☐ Antibodies
☐	☒ Eukaryotic cell lines
☒	☐ Palaeontology and archaeology
☒	☐ Animals and other organisms
☒	☐ Clinical data
☒	☐ Dual use research of concern

Methods

n/a	Involved in the study
☒	☐ ChIP-seq
☒	☐ Flow cytometry
☒	☐ MRI-based neuroimaging

Eukaryotic cell lines

Policy information about cell lines and Sex and Gender in Research

Cell line source(s)	All strains used in this study were derived from the wild type yeast (Saccharomyces cerevisiae) strain CEN.PK2-1C (EuroSCARF3 0000A).
Authentication	All yeast strains with chromosomal editing were validated by genotyping PCR and sequencing of the modified loci.
Mycoplasma contamination	Not applicable.
Commonly misidentified lines (See ICLAC register)	Not applicable.
